# Pentosan polysulfate sodium prevents functional decline in chikungunya infected mice by modulating growth factor signalling and lymphocyte activation

**DOI:** 10.1371/journal.pone.0255125

**Published:** 2021-09-07

**Authors:** Penny A. Rudd, Elisa X. Y. Lim, Catherine J. M. Stapledon, Ravi Krishnan, Lara J. Herrero

**Affiliations:** 1 Institute for Glycomics, Griffith University, Southport, Qld, Australia; 2 Paradigm Biopharmaceuticals Ltd, Melbourne, Australia; CEA, FRANCE

## Abstract

Chikungunya virus (CHIKV) is an arthropod-borne virus that causes large outbreaks world-wide leaving millions of people with severe and debilitating arthritis. Interestingly, clinical presentation of CHIKV arthritides have many overlapping features with rheumatoid arthritis including cellular and cytokine pathways that lead to disease development and progression. Currently, there are no specific treatments or vaccines available to treat CHIKV infections therefore advocating the need for the development of novel therapeutic strategies to treat CHIKV rheumatic disease. Herein, we provide an in-depth analysis of an efficacious new treatment for CHIKV arthritis with a semi-synthetic sulphated polysaccharide, Pentosan Polysulfate Sodium (PPS). Mice treated with PPS showed significant functional improvement as measured by grip strength and a reduction in hind limb foot swelling. Histological analysis of the affected joint showed local inflammation was reduced as seen by a decreased number of infiltrating immune cells. Additionally, joint cartilage was protected as demonstrated by increased proteoglycan staining. Using a multiplex-immunoassay system, we also showed that at peak disease, PPS treatment led to a systemic reduction of the chemokines CXCL1, CCL2 (MCP-1), CCL7 (MCP-3) and CCL12 (MCP-5) which may be associated with the reduction in cellular infiltrates. Further characterisation of the local effect of PPS in its action to reduce joint and muscle inflammation was performed using NanoString™ technology. Results showed that PPS altered the local expression of key functional genes characterised for their involvement in growth factor signalling and lymphocyte activation. Overall, this study shows that PPS is a promising treatment for alphaviral arthritis by reducing inflammation and protecting joint integrity.

## Introduction

Chikungunya virus (CHIKV) is an arthropod-borne virus belonging to the *Togaviridae* family and alphavirus genus which is transmitted via the *Aedes* species of mosquitoes. Initial signs of infection include high fever and chills, severe arthralgia and myalgia, maculopapular rash, general weakness and headache. Painful rheumatic manifestations often affect large joints (hands and feet) in a symmetrical manner. In a majority of cases, the debilitating polyarthritis lasts more than 3 months and can last up to several years post-infection [1, 2].

Interestingly, the chronic arthritis that ensues after the initial CHIKV infection can be clinically mistaken for rheumatoid arthritis (RA). As in RA, CHIKV commonly affects the metacarpophalangeal, proximal interphalangeal, and wrist joints as well as knees and ankles [3, 4]. Furthermore, other symptoms described by RA patients with accompanying fibromyalgia are also experienced by CHIKV patients. These include bursitis, tendonitis, joint oedema, myalgias and morning stiffness [2]. Magnetic resonance imaging (MRI) has shown that both conditions cause joint effusions, bony erosions and synovial thickening, all of which contribute to the joint degeneration and pain commonly observed and experienced in CHIKV patients [5].

Over the last fifteen years, CHIKV has received significant attention due to its unprecedented spread and repetitive large outbreaks, including the most recent Caribbean outbreak where all islands were affected and over 800 000 cases (2% of the population) were diagnosed. Since then, local transmission has been identified in 45 countries or territories in the Americas with more than 2.9 million cases reported. Due to global vector dissemination, climate change and increased international travel, CHIKV is expected to continue to spread to new regions posing an important public health burden [6].

Along with the significant medical burden associated with CHIKV infections, there are also considerable economic costs of widespread epidemics. Up to 79% of patients will suffer from chronic arthralgia and inflammatory rheumatism, resulting in decreased quality of life for months to years following initial infection [7, 8]. Cost of illness studies for the most recent CHIKV outbreaks (2005–2006 Reunion Island and 2014–2015 Virgin Islands) were between €27.8 million and €43.9 million [9, 10]. These costs were inclusive of both direct and indirect expenses such as time off work, healthcare visits, hospitalisations and pharmaceutical treatment.

To date, there are no licensed vaccines or specific therapeutics available for the treatment of CHIKV arthritides. During active infection, treatment strategies consist mainly of supportive care including the administration of fluids, analgesics and antipyretics. Management of the debilitating pain caused by CHIKV includes taking acetaminophen, codeine and/or neuropathic pain medications like gabapentin [11]. Non-steroidal anti-inflammatory drugs (NSAIDs) can also alleviate the inflammatory aspects of sub-acute and chronic disease however, long-term use of NSAIDs has unwanted side effects including gastric ulcers, cardiovascular complications, and renal impairment. Systemic corticosteroids are only recommended for patients presenting with chronic highly inflammatory polyarthritis but their use has been shown to increase the risk of developing infection, cataracts, glaucoma, diabetes and osteoporosis [12] and are contraindicated in acute CHIKV disease [13]. Given the extensive burden of CHIKV-induced rheumatic conditions and the complications of non-specific treatments currently available, there is an urgent need for a targeted therapeutic agent. Towards this, we have recently described a novel glycosaminoglycan (GAG)-like molecule, Pentosan Polysulfate Sodium (PPS) for effective treatment of alphavirus-induced arthritides as a result of Ross River virus (RRV) infection [14, 15]. Herein, we further characterised the efficacy of PPS towards the treatment of the global CHIKV-induced disease and explored clinically significant pathways altered by PPS treatment.

In this study, we used a well-established adult mouse model of CHIKV infection that recapitulates many key features of human CHIKV disease [16]. Our results show that treatment with PPS significantly reduced clinical signs of disease and improved functional strength. Treatment with PPS reduced the amount of infiltrating cells and protected the joints from cartilage damage. Using NanoString™ technology, we have shown that PPS modulates several pathways including growth factor signalling and lymphocyte activation that contribute to the reduction of inflammation in CHIKV-induced arthralgia. Overall, this study provides insights into the mechanism of PPS treatment in CHIKV infection through reducing inflammation and improving clinical outcome.

## Materials and methods

### Viruses and cells

Stocks of the Réunion Island CHIKV isolate LR2006-OPY1, a distinct clade within the East/Central/South Africa (ECSA) genotype, were propagated in C6/36 (ATCC® CRL-1660™) cells from a full-length cDNA clone, kindly provided by A. Merits, as previously described [17]. All titrations were performed by plaque assays on Vero cells as described previously [18].

### Mouse infections and PPS treatment

All animal experiments were conducted in strict accordance with the *Australian Code for the Care and Use of Animals for Scientific Purposes* and this study was approved in writing by the Animal Ethics Committee of Griffith University under the permit; GLY/15/19. Female C57BL/6 wild-type (WT) mice were obtained from the Animal Resources Centre (Perth, Australia). As previously described, mice were inoculated with 10^4^ plaque forming units (PFU) of LR2006-OPY1 CHIKV subcutaneously (s.c.) in the metatarsal region of the dorsal side of both hind feet, injecting toward the ankle [19]. Mock-infected mice were inoculated s.c. with vehicle comprising of endotoxin free phosphate buffered saline (PBS) alone. Treatment with PPS (Fibrase) 100 mg/ml, (Teofarma, Valle Salimbene, IT) or vehicle alone (endotoxin free PBS) was given intraperitoneally (i.p.) at 3 mg/kg of body weight in 100 μl daily for the duration of the experiment, commencing 4 hours prior to virus infection. Upon termination of the experiment, euthanasia was carried out humanely using carbon dioxide exposure and death was verified by the absence of both respiration and heartbeat prior to tissue collection.

### Clinical disease measurements

Every 24h, mice were weighed and scored for signs of disease. Signs of clinical disease determined by footpad swelling was monitored by measuring the height and width of the metatarsal area of the hind feet using digital callipers.

### Grip strength measurements

Grip strength of all limbs was measured daily with a validated computerized grip strength meter (model BIO-GS3, BIOSEB SL, Vitrolles, France). The apparatus consisted of a grid connected to a force transducer. To evaluate grip strength of all paws, mice were placed over the grid until paws grasped the grid. The peak force of each measurement was automatically recorded in grams (g) by the device. Limb grip strength for each mouse was measured in triplicate and readings were recorded and averaged. Grip strength was also recorded the day prior to the commencement of the experiment to assess for baseline value of strength. This value was considered as 100% of grip strength and used as a reference for subsequent determinations. Change in grip strength was determined by calculating the absolute strength increase over a time period (Force Time x–Force Time 0) normalised to body weight (Force Time x/body weight) and where FT0 represents the baseline value of strength (pre-infection) [20, 21].

### Chemokine and cytokine analyses

Serum chemokine and cytokine protein levels were determined by using the Bio-Plex Pro™ Mouse Chemokine 33-plex bead array kit according to the manufacturer’s instructions (Bio-Rad, Hercules, CA). Data were acquired using a Bio-Plex 200^®^ instrument (Bio-Rad) and analysed with the Bio-Plex Manager software version 6.1.

### Histology

Tissues were fixed in 4% paraformaldehyde and hind limbs were decalcified (15% EDTA in 0.1% phosphate buffer over 10 days). Subsequently, tissue samples were embedded in paraffin wax, and 5-μm-thick sections were cut and stained with hematoxylin-eosin (H&E) or Safranin O (Saf’O). Slides were scanned using an Aperio Scan Scope XT digital slide scanner (Aperio, Vista, CA, USA). The tissues from all groups were evaluated by light microscopy for any evidence of histopathological changes by a veterinary pathologist blinded to treatments and infection status. Changes in cartilage were scored as follows: grade 0 = within normal limits/no change, grade 1 = minimal depletion of sulfated GAGs, grade 2 = mild depletion of sulfated GAGs, grade 3 = moderate depletion of sulfated GAGs with signs of cartilage shrinkage, grade 4 = marked/severe depletion of sulfated GAGs with clear cartilage shrinkage. Changes in bone were scored as follows: grade 0 = within normal limits/no change, grade 1 = minimal change in bone necrosis, grade 2 = mild change in bone necrosis with observed changes in osteoclast/osteoblast ratios, grade 3 = moderate change in bone necrosis with observed changes in osteoclast/osteoblast ratios and/or vascular changes, grade 4 = marked/severe change in bone necrosis with clear changes in osteoclast/osteoblast ratios and/or strong vascular changes.

### RNA isolation and nanostring™ nCounter® gene expression profiling

RNA was extracted from ankle joints and quadriceps using 1 ml and 0.5 ml respectively of TRIzol™ reagent (Invitrogen, Carlsbad, CA) according to the manufacturer’s instructions. The quality of the RNA was assessed on a LabChip GX touch (Perkin Elmer) and quantified using the Promega QuantiFluor RNA system® as per instructions. Gene expression analysis of RNA was performed using the commercially available NanoString™ nCounter® mouse Myeloid Innate Immunity gene expression panel (NanoString™ Technologies, Seattle, WA, USA) following the manufacturer’s instructions. This panel contains 20 internal reference genes for data normalisation and 754 target genes including several known to be regulated during CHIKV infection. Raw gene expression data was normalised against a set of positive and negative controls to account for background noise and platform associated variation. Reference gene normalisation was performed using the GeNorm Algorithm where housekeeping genes were selected based on the lowest variance across samples.

### Protein-Protein Interaction (PPI) network

The STRING database (http://string-db.org/) [22] was used to identify the interactions between the top DEGs modulated during PPS treatment of CHIKV-infected animals. Top genes chosen had a fold change (FC) >1.3 or FC < -1.3 and a P value < 0.02. Each node represents a gene and the connections between nodes represent the interaction of these biological molecules, which can be used to identify interactions and pathway relationships between the proteins encoded by DEGs in PPS treatment of CHIKV. Furthermore, Kyoto Encyclopedia of Genes and Genomes (KEGG) pathway enrichment analysis was also performed and the top 5 pathways with the smallest false discovery rates (FDR) were compiled. Further analysis using the REACTOME database revealed the top 5 biological pathways involved. NanoString™ also provide annotations to their panels which allows for sorting of key genes by functional group. Key DEGs were sorted using these annotations and the top 3 functional groups were reported.

### Statistics

Data for multiplex bead array, foot swelling, and absolute grip strength (normalised to body weight over time) were analysed using a One-Way analysis of variance (ANOVA) with Tukey’s post-test. Data for normalised grip strength was analysed using a Two-Way ANOVA and Sidak’s multiple comparison test. Histological analysis was performed using a student t-test correction. For the gene expression analysis, Limma package was used [23] and P values were adjusted for multiple testing by the Benjamini and Hochberg method to control the false discovery rate [24]. Statistics were performed with GraphPad Prism 8.3.1.

## Results

### PPS treatment of CHIKV in mice improves grip strength and foot swelling

We have recently reported that PPS is able to improve hand strength in patients suffering from RRV [15]. By using a well characterised adult mouse model of CHIKV infection [16], we assessed if PPS treatment could treat the functional signs of CHIKV disease by improving grip strength. Mice were either mock-infected with PBS alone (‘mock’), mock-infected, PPS-treated (‘PPS alone’), CHIKV-infected mock-treated (‘CHIKV-infected untreated’) or CHIKV-infected, PPS-treated (‘CHIKV-infected PPS-treated’). All CHIKV infections were accomplished by giving 10^4^ PFU/hind foot and all PPS treatments consisted of injecting PPS i.p. at a dose of 3 mg/kg daily for either 7 days (peak disease, n = 15) or 21 days (disease resolution, n = 5). Grip strength was assessed in triplicate measurements per mouse, daily.

**Fig 1:**
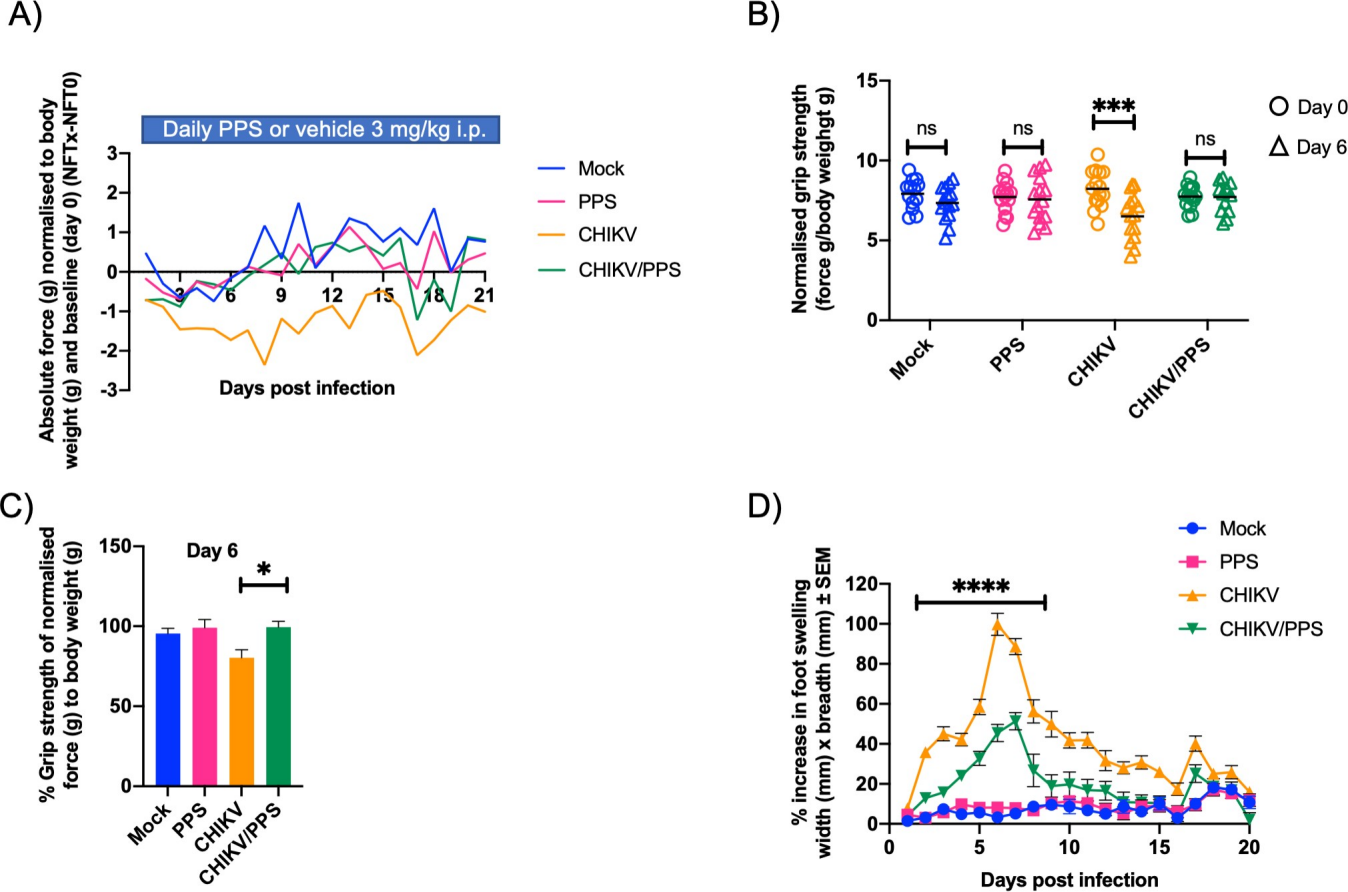
Treatment of CHIKV-infected mice with PPS improves grip strength and hind-foot swelling. C57BL/6 mice were infected s.c. with 10^4^ PFU CHIKV or PBS alone and received daily injections of PPS or mock-treatment with PBS. Mice were measured daily (in triplicate) for grip strength and joint swelling (both hind feet). All data are presented as mean ± SEM, n = 15 mice from days 0–7 and n = 5 mice from days 8–21 post-infection. Mock-infected; mock, mock-infected PPS-treated; PPS, CHIKV-infected mock-treated; CHIKV, CHIKV-infected PPS-treated; CHIKV/PPS. **(A)** Grip strength described as absolute force (g) normalised to body weight (g) and compared to baseline (day 0) (NFTx—NFT0). Significant differences (****P < 0.0001) were observed between CHIKV-infected untreated mice and all other groups, CHIKV-infected untreated; vs mock, vs PPS alone, and vs CHIKV-infected PPS-treated. *One-Way ANOVA*, *Tukey’s post-test* **(B)** Normalised grip strength described as force (g)/body weight (g) at days 0 and 6 post-infection. Grip strength was significantly reduced between baseline (day 0; 8.2 ± 0.3) and peak disease (day 6; 6.5 ± 0.4) in the CHIKV-infected untreated group only (***P < 0.0002). All other groups displayed no significant differences in grip strength between days 0 and 6 post-infection, *Two-Way ANOVA and Sidak’s multiple comparisons test*. **(C)** Percentage change in normalised grip strength [force (g)/body weight (g)] between 0 and day 6 post-infection. At 6 d.p.i., CHIKV-infected untreated mice lost 19.8% ± 5.1 of their original strength compared to a 0.5% ± 3.6 reduction for the CHIKV-infected PPS-treated mice. There were no statistically significant changes observed between mock, PPS alone and CHIKV-infected PPS-treated groups. *One-Way ANOVA with a Tukey’s post-test*. **(D)** CHIKV-induced joint swelling measured daily in triplicate and displayed as percentage increase compared to baseline. Significant reductions in swelling were observed between the CHIKV-infected untreated and CHIKV-infected PPS-treated mice between 2 and 11 d.p.i. and 13 and 14 d.p.i. P values were: 2 d.p.i. to 9 d.p.i. (****P < 0.0001), 10 d.p.i. (**P = 0.0039); 11 d.p.i. (***P = 0.0007); 13 d.p.i. (*P = 0.0399), and 14 d.p.i. (*P = 0.0108). *Two-Way ANOVA with a Tukey’s post-test*.

CHIKV-infected untreated animals demonstrated a decrease in limb strength from baseline from 3 to 8 days post-infection (d.p.i.) (****P < 0.0001), as shown by normalised strength over time (NFTx–NFT0) (Fig 1A). At 3 d.p.i. (the onset of swelling) CHIKV-infected untreated mice lost 16% ± 5.8 (mean ± SEM) of their original strength whereas CHIKV-infected PPS-treated animals had only a marginal decrease of 7.8% ± 4.9. At 8 d.p.i., CHIKV-infected untreated mice had a 21.5% reduction of their original strength whereas CHIKV-infected PPS-treated animals had an increase of strength over baseline of 10.9% ± 5.3 (Fig 1A).

Mock, PPS alone and CHIKV-infected PPS-treated animals displayed improved grip strength over the course of the experiment. CHIKV-infected PPS-treated improved by 11.4% ± 5.4, mock by 22.8% ± 13.5 and PPS alone by 3.5% ± 4.9. At the conclusion of the experiment, CHIKV-infected untreated mice had not recovered complete strength displaying a loss of 7.8% ± 10.5.

Comparing the differences in grip strength between groups, there were no observable changes between the mock and PPS alone groups throughout the experiment (Fig 1A). CHIKV-infected untreated animals showed significantly reduced strength from mock, PPS alone and CHIKV-infected PPS-treated animals (****P < 0.0001) (Fig 1A), throughout the experiment.

Analysis of normalised grip strength [force (g)/body weight (g)] at baseline (day 0) and peak disease (day 6) did not show any significant changes in the mock, PPS alone or CHIKV-infected PPS-treated groups (Fig 1B). However, the CHIKV-infected untreated group showed a significant reduction (***P < 0.0002) in normalised grip strength at peak disease (6.5 ± 0.4; mean ± SEM) compared to baseline values (8.2 ± 0.3). This equated to an overall 19.8% ± 5.1 reduction in grip strength in the CHIKV-infected untreated group between 0 and 6 d.p.i. (Fig 1C).

In the CHIKV-infected PPS-treated mice, grip strength was unchanged between days 0 and 6 post-infection (reduction of 0.5% ± 3.6, mean ± SEM). When CHIKV-infected untreated and CHIKV-infected PPS-treated groups were compared, a significant difference in the percentage change between 0 and 6 d.p.i. was shown (*P < 0.02) (Fig 1C). It is important to note that all groups saw no significant difference in weight changes throughout the duration of the experiment (S1 Fig).

To assess disease severity, hind foot swelling [width (mm) x breadth (mm)] was assessed daily in both peak disease (6–7 d.p.i.) and disease resolution (21 d.p.i.). Mock animals had marginal increases of 3.2% ± 2.0; mean ± SEM (6 d.p.i.) and 5.2% ± 1.9 (7 d.p.i.). PPS alone had an increase of 8.2% ± 1.7 (6 d.p.i.) and 7.9% ± 1.5 (7 d.p.i.). Both of which are attributed to normal mouse growth over time.

Our results were consistent with Gardner *et al*. [16], with peak CHIKV disease seen at 6–7 d.p.i. as indicated by the significant increase in foot swelling (Fig 1D). CHIKV-infected untreated mice had an increase from baseline of 99.7% ± 5.6; mean ± SEM (6 d.p.i.); and 88.6% ± 4.0 (7 d.p.i.). CHIKV-infected PPS-treated animals only showed an increase of 45.4% ± 4.3 (6 d.p.i.) and 51.3% ± 4.3 (7 d.p.i.). This represented a significant reduction in swelling between CHIKV-infected untreated and CHIKV-infected PPS-treated mice (****P < 0.0001).

Swelling was overall significantly different between CHIKV-infected untreated and CHIKV-infected PPS-treated groups between days 2 and 11 post-infection and days 13 and 14 post-infection (Fig 1D). Significant differences were also observed between the CHIKV-infected untreated group compared to both mock and PPS alone (****P < 0.0001) (Fig 1D).

### PPS reduces the number of infiltrates in the hind limbs at peak infection

**Fig 2:**
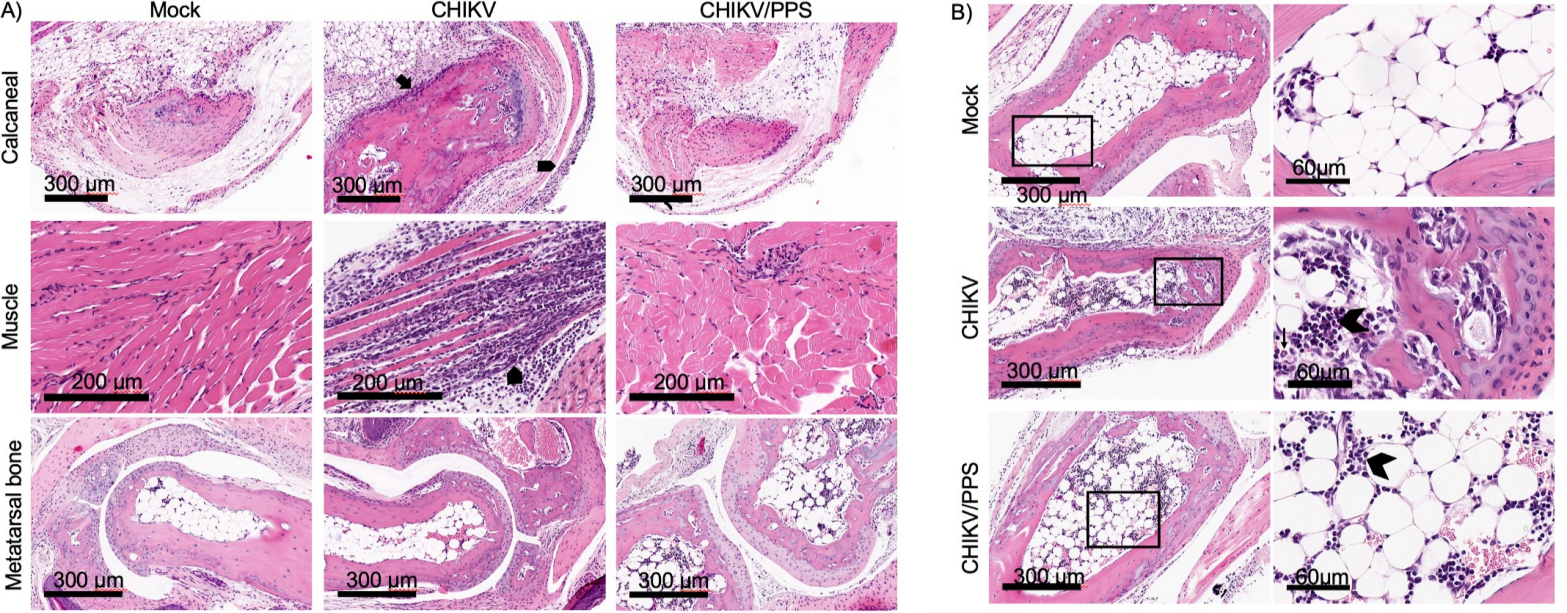
Histological analysis of PPS-treated mice at peak disease. C57BL/6 mice were infected s.c. with 10^4^ PFU CHIKV or PBS alone and received daily injections of PPS-treatment or mock with PBS. Mice were sacrificed at 7 d.p.i. and tissues collected and fixed for histological analysis. **(A)** H&E staining of the hind limbs of CHIKV-infected mice. Increase in cellular infiltrates were seen in the calcaneal region, the muscle, and the tissue adjacent to the metatarsal bones in CHIKV-infected untreated mice. CHIKV-infected PPS-treated mice showed a reduction in inflammatory cells when compared to CHIKV-infected untreated mice. Scale bars represent 200 μm (muscle) and 300 μm (calcaneal and bone). **(B)** Infiltrating cells can also be found in the bone marrow. Again, CHIKV-infected PPS-treated mice showed a reduction in infiltrates when compared to CHIKV-infected untreated mice. Scale bars represent 60 μm and 300 μm. All slides were scanned with the Aperio Scan Scope XT digital slide scanner. Images are representative of 5 mice per group, 2 sections per mouse. Mock-infected; mock, CHIKV-infected mock-treated; CHIKV, CHIKV-infected, PPS-treated; CHIKV/PPS.

Histological analysis was conducted to assess the effects of PPS on local inflammation following CHIKV infection. Tissues were collected at both peak disease (7 d.p.i.) and upon resolution of infection (21 d.p.i.). H&E staining of mock and PPS alone treatment groups displayed no observable inflammation (Fig 2A). Abundant infiltrates characteristic of monocytes and neutrophils were seen in the calcaneal region, surrounding muscle, metatarsal bones, and bone marrow in the CHIKV-infected untreated group (Fig 2A and 2B). In contrast, CHIKV-infected PPS-treated mice displayed a visible reduction in the overall number of infiltrates in these structures of the hind limbs. Interestingly, at day 21, histological analyses showed complete disease resolution. The number of infiltrating cells between mouse groups did not differ significantly. However, treatment of PPS protected muscle fibres from damage (S2 Fig). Furthermore, PPS treatment appeared to accelerate the inflammatory repair processes with evidence of an increase in the number of regenerating myocytes (S3 Fig). Additionally, the reduction in clinical disease score and joint inflammation was not a result of reduced viral load in CHIKV-infected PPS-treated mice (S4 Fig).

### PPS treatment reduces joint destruction

**Fig 3:**
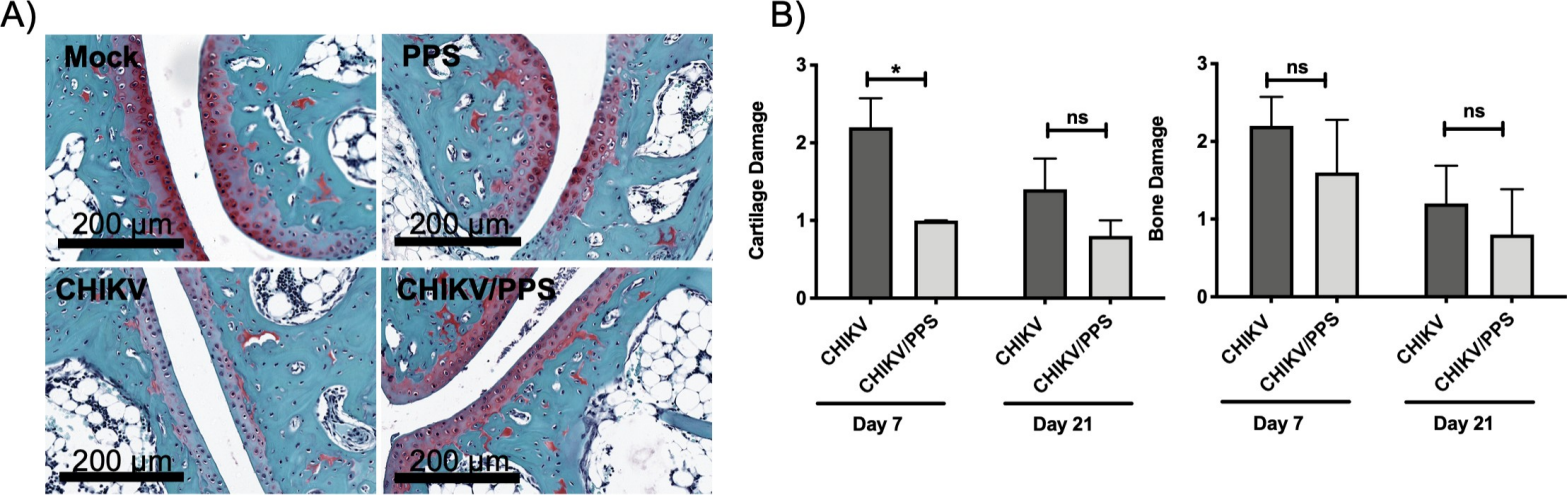
Safranin O staining of articular cartilage in the calcaneal joint. C57BL/6 mice were infected s.c. with 10^4^ PFU CHIKV or PBS alone and received daily injections of PPS-treatment or mock-treatment with PBS. Mice were sacrificed at 7 d.p.i. and ankle joint including foot collected, decalcified and fixed for safranin O staining. **(A)** CHIKV-infected PPS-treated mice showed less depletion of sulfated glycosaminoglycans compared to CHIKV-infected untreated mice. High magnification images were taken at peak disease (7 d.p.i.). Slides were scanned with the Aperio Scan Scope XT digital slide scanner. Scale bars represent 200 μm. Images are representative of 5 mice per group. **(B)** The extent of cartilage and bone damage was determined by scoring for histopathological changes with sample identity blinded to the reader. To assess cartilage damage, changes in cartilage were scored 0–4 ranging from within normal limits to severe depletion of sulfated glycosaminoglycans and cartilage shrinkage [7 d.p.i. CHIKV-infected untreated 2.2 ± 0.4 vs 1.0 ± 0.002 CHIKV-infected PPS-treated (*P = 0.0125)]. Bone damage was scored 0–4 ranging from within normal limits to severe osteoclast/osteoblast activity, bone necrosis and vascular changes. No statistical significance was seen between CHIKV-infected untreated and CHIKV-infected PPS-treated groups (n = 5 animals/group). *Student t-test correction*.

Saf-O staining was performed to assess the integrity of the articular cartilage and bone pathology. Saf-O staining is directly proportional to the amount of proteoglycan content in cartilage and can therefore indicate a disease state. Representative images of Saf-O staining are shown in Fig 3A. CHIKV-infected untreated mice showed a marked depletion of sulfated GAGs (i.e., decrease in Saf-O staining) with corresponding cartilage shrinkage (Fig 3A), which was significantly improved with PPS treatment (*P = 0.0125, Fig 3B). Changes in cartilage (Fig 3B) were blindly assessed in a semi-quantitative manner using a scale of 0–4, 4 being the most severe. CHIKV-infected untreated mice had a score of 2.2 ± 0.4 (mean ± SEM) on day 7 p.i. and 1.4 ± 0.4 on day 21 post-infection. In comparison, CHIKV-infected PPS-treated mice had less severe cartilage changes 1.0 ± 0.002 on day 7 p.i. and 0.8 ± 0.2 on day 21 post-infection. Mice from mock and PPS alone groups did not show any changes in cartilage and scored 0 (n = 5 mice/group).

It has been reported that CHIKV infection results in bone damage, with bone necrosis driven by increased osteoclast activity [25]. Our results confirm CHIKV infection results in bone damage, with bone damage alone marginally improved (non-significant) in CHIKV-infected PPS-treated mice (Fig 3B). Like for cartilage, changes in bone were assessed on a scale of 0–4, 4 being the most severe. CHIKV-infected untreated mice scored 2.2 ± 0.4 on day 7 p.i. and 1.2 ± 0.5 on day 21 post-infection. CHIKV-infected PPS-treated mice scored 1.6 ± 0.7 on day 7 p.i. and 0.8 ± 0.6 on day 21 post-infection. Control groups mock and PPS alone scored 0 (n = 5 mice/group). Taken together, the results show PPS treatment protects joint cartilage but not bone during CHIKV infection.

### PPS treatment modifies the serum levels of chemokines and cytokines in CHIKV-induced inflammation

**Fig 4:**
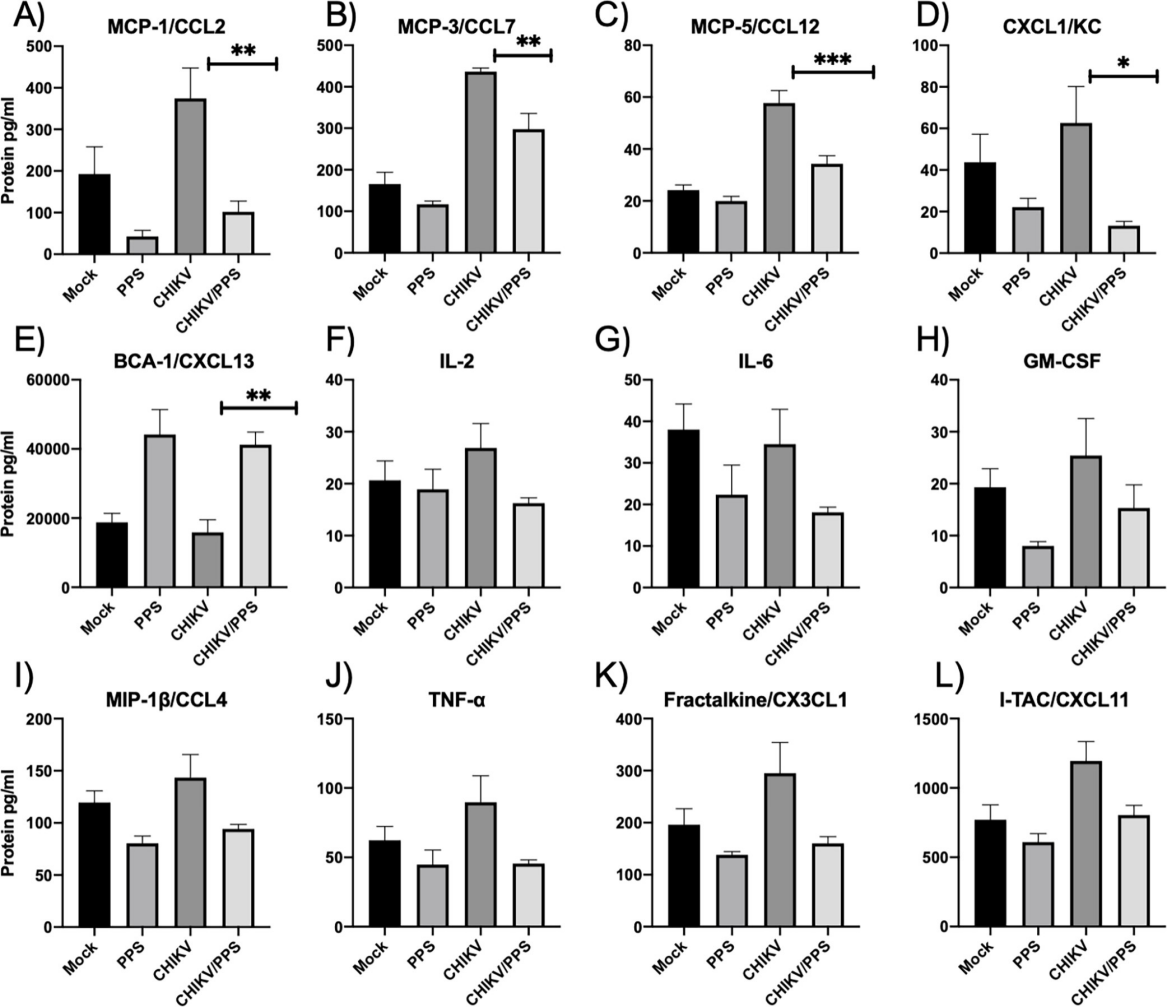
Effect of pentosan polysulfate sodium on the serum levels of chemokines and cytokines involved in inflammation. Chemokine and cytokine levels of mock, PPS alone (PPS), CHIKV-infected untreated (CHIKV) and CHIKV-infected PPS-treated (CHIKV/PPS) mice were assessed at peak disease (7 d.p.i.). All values are presented as mean pg/mL ± SEM of 5 mice per group. Expression was significantly reduced between CHIKV-infected untreated and CHIKV-infected PPS-treated groups. **(A)** CCL2 (101.8 ± 25.9 vs 374.6 ± 73, **P = 0.058). **(B)** CCL7 (297.9 ± 37.6 vs 436.5 ± 8.9 **P = 0.0047) **(C)** CCL12 (34.3 ± 3.1 vs 57.7 ± 4.9 ***P = 0.0007) and **(D)** CXCL1 (13.2 ± 2.1 vs 62.6 ± 17.7 *P = 0.0331). **(E)** CXCL13 expression was significantly increased between CHIKV-infected untreated and CHIKV-infected PPS-treated groups (41239.5 ± 3612.3 vs 15868.6 ± 3636.2 **P = 0.0080)). *One-Way ANOVA with a Tukey’s post-test*. **(F-L)** Chemokines/cytokines that displayed a decreased trend in protein expression without meeting statistical significance are also shown.

Serum chemokine and cytokine levels of all groups were assessed at 7 d.p.i. (peak disease) (Fig 4). As previously described, CHIKV infection alters soluble factors including up-regulating CCL2 and TNF-α compared to mock controls [16, 26]. Compared to CHIKV-infected untreated mice, CHIKV-infected PPS-treated mice showed significant reductions in serum biomarkers for the chemokines CCL2 (**P = 0.058), CCL7 (**P = 0.0047), CCL12 (***P = 0.0007) and the chemokine (C-X-C motif) ligand 1 (CXCL-1; *P = 0.0331). See Fig 4 for all values. In contrast, B cell-attracting chemokine 1 (CXCL13; BCA-1) was upregulated (**P = 0.0080) in CHIKV-infected PPS-treated group compared to the CHIKV-infected untreated group (Fig 4A–4E). We also investigated a range of other chemokines and cytokines, which were not significantly altered but were trending towards a reduction in protein expression after treatment with PPS (Fig 4F–4L). In particular, we observed this downward trend for interlukin-2 (IL-2), interleukin-6 (IL-6), granulocyte-macrophage colony-stimulating factor (GM-CSF), macrophage inflammatory 1 protein-1 beta (MIP-1β; CCL4), TNF-α, CX3CL1 (fractalkine), and interferon-inducible T-cell alpha chemoattractant (CXCL11; I-TAC;). Twenty-one chemokines/cytokines in the 33-plex panel showed no significant differences or trends (S5 Fig).

### PPS modulates specific pathways in both joint and muscle tissues during CHIKV infection

Using the NanoString™ nCounter® technology, we observed modulation of a range of key inflammatory genes when comparing CHIKV-infected untreated mice to mock control mice (S6 Fig, S1 and S2 Tables). Pathway modulation during CHIKV infection was indicated by the upregulation of key genes, several of which are consistent with previous reports on CHIKV infection in humans and mice [27]. These key upregulated genes included chemokines *Cxcl10*, *Cxcl9*, C-C chemokine receptor type 5 (*Ccr5*), CC chemokine ligand 2 (*Ccl2*), *Ccl4*, *Ccl7*, *Ccl8* and *Ccl12* as well as T-cell associated genes such as transporter associated with antigen processing 1 (*Tap1*) and histocompatibility 2, T region locus 23 (*H2-T23*), and sensing/signalling effectors like interferon regulatory factor 7 (*Irf7*) and signal transducer and activator of transcription 1 (*Stat1*). In addition, a three-fold increase and **P value of ≤ 0.01 in gene expression of interferon-induced GTP-binding protein Mx1 (*Mx1*), and granzyme A (*Gzma*) was also observed.

**Fig 5:**
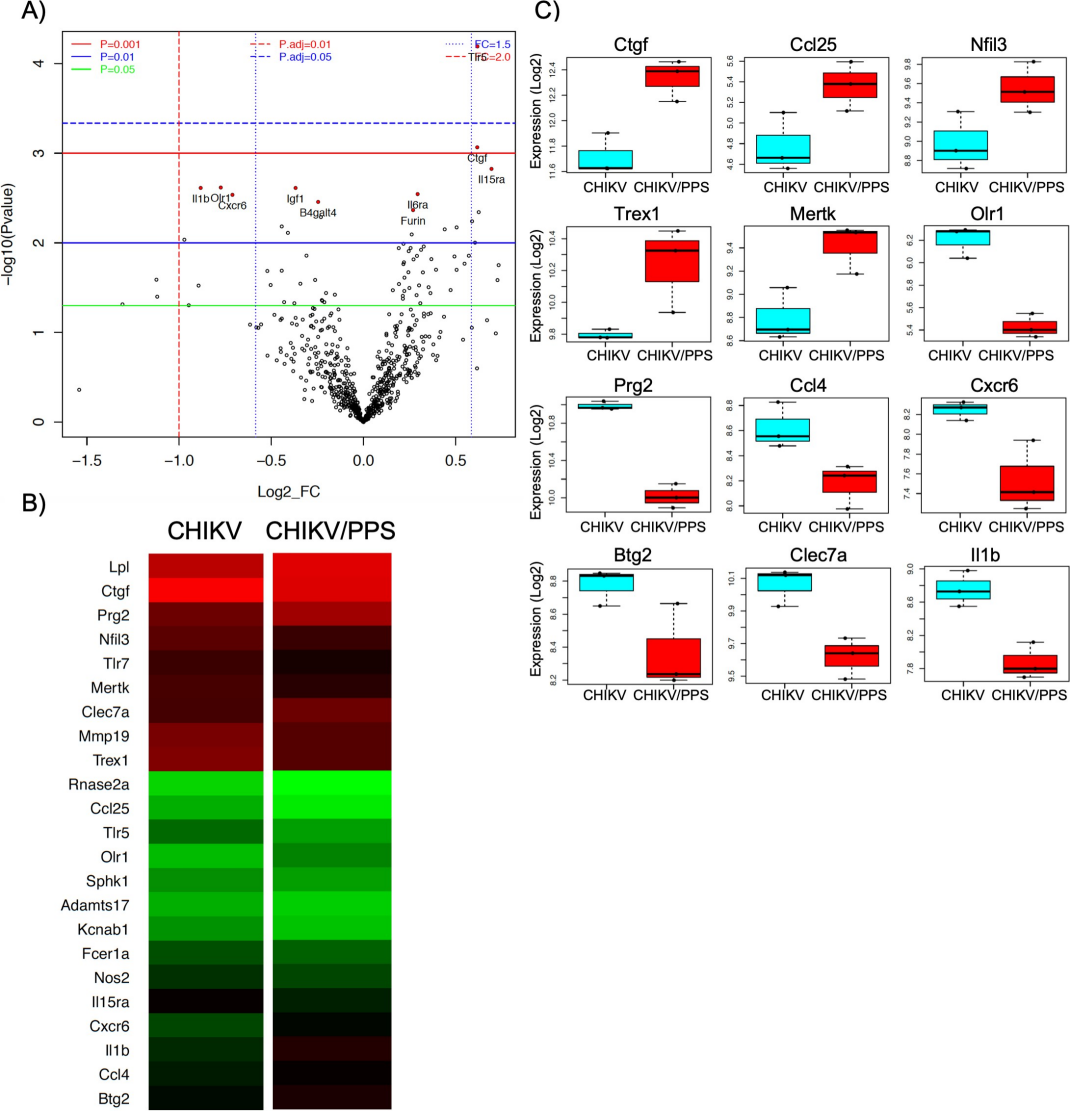
qPCR NanoString™ panel and bioinformatic analysis of joint tissue. Gene expression analysis of RNA isolated from joint tissue at peak disease was performed using the commercially available NanoString™ nCounter® mouse Myeloid Innate Immunity gene expression panel. Data shows **(A)** volcano plots, **(B)** heat maps and **(C)** box plots (Log2) of key genes that are modulated in mouse joints during PPS treatment. Gene expression data was analysed using Limma package and P values were adjusted for multiple testing by Benjamini and Hochberg method to control the false discovery rate. Data compares tissues from CHIKV-infected PPS-treated mice to CHIKV-infected untreated mice (n = 3 mice/group). Top genes chosen had a FC >1.3 or FC < -1.3 and a P value < 0.02.

Analysis of genes that were differentially expressed between CHIKV-infected untreated and CHIKV-infected PPS-treated groups, identified several DEGs known to contribute to the pathobiology of arthritis. Such DEGs included connective tissue growth factor (*Ctgf*), cyclin-dependent kinase inhibitor 1 (*Cdkn1a*), nuclear factor IL-3 regulated (*Nfil3*), *Stat1*, fatty acid-binding protein 4 (*Fabp4*), cAMP response element modulator (*Crem*), c-type lectin domain family 7 member A (*Clec7a*; *Dectin-1*), *Il-1β*, proto-oncogene tyrosine-protein kinase MER (*Mertk*), three prime repair exonuclease 1 (*Trex1*), B-cell translocation gene 2 (*Btg2*), serine protease inhibitor clade E member 1 (*Serpine1*), *Ccl25* and oxidized low-density lipoprotein receptor 1 (*Olr1*), as indicated by volcano plots, heat maps and box plots [Figs 5 (joint) and 6 (muscle)]. All factors had at least a 1.3-fold change (FC) and a *P value ≤ 0.02. Interestingly, the top genes in joint and muscle tissues differed except for *Nfil3* and Fc fragment of IgE, high affinity I receptor for alpha polypeptide (*Fcer1a*) (S3 and S4 Tables).

**Fig 6:**
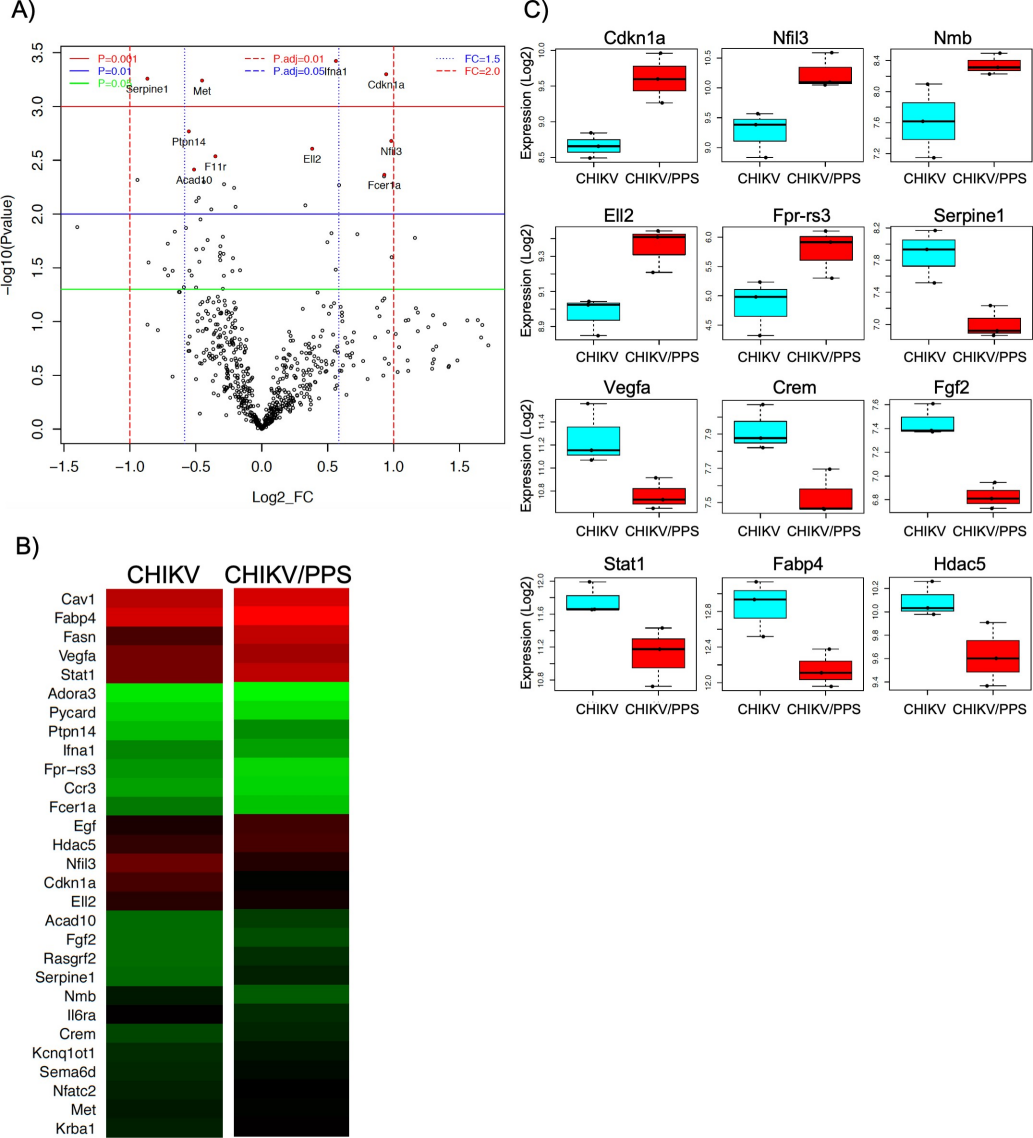
qPCR NanoString™ panel and bioinformatic analysis of muscle tissue. Gene expression analysis of RNA isolated from muscle tissue at peak disease was performed using the commercially available NanoString™ nCounter® mouse Myeloid Innate Immunity gene expression panel. Data shows **(A)** volcano plots, **(B)** heat maps and **(C)** box plots (Log2) of key genes which are modulated in quadriceps during PPS treatment. Gene expression data was determined using Limma package and P values were adjusted for multiple testing by Benjamini and Hochberg method to control the false discovery rate. Data compares tissues from CHIKV-infected PPS-treated mice to CHIKV-infected untreated mice (n = 3 mice/group). Top genes chosen had a FC >1.3 or FC < -1.3 and a P value < 0.02.

**Fig 7:**
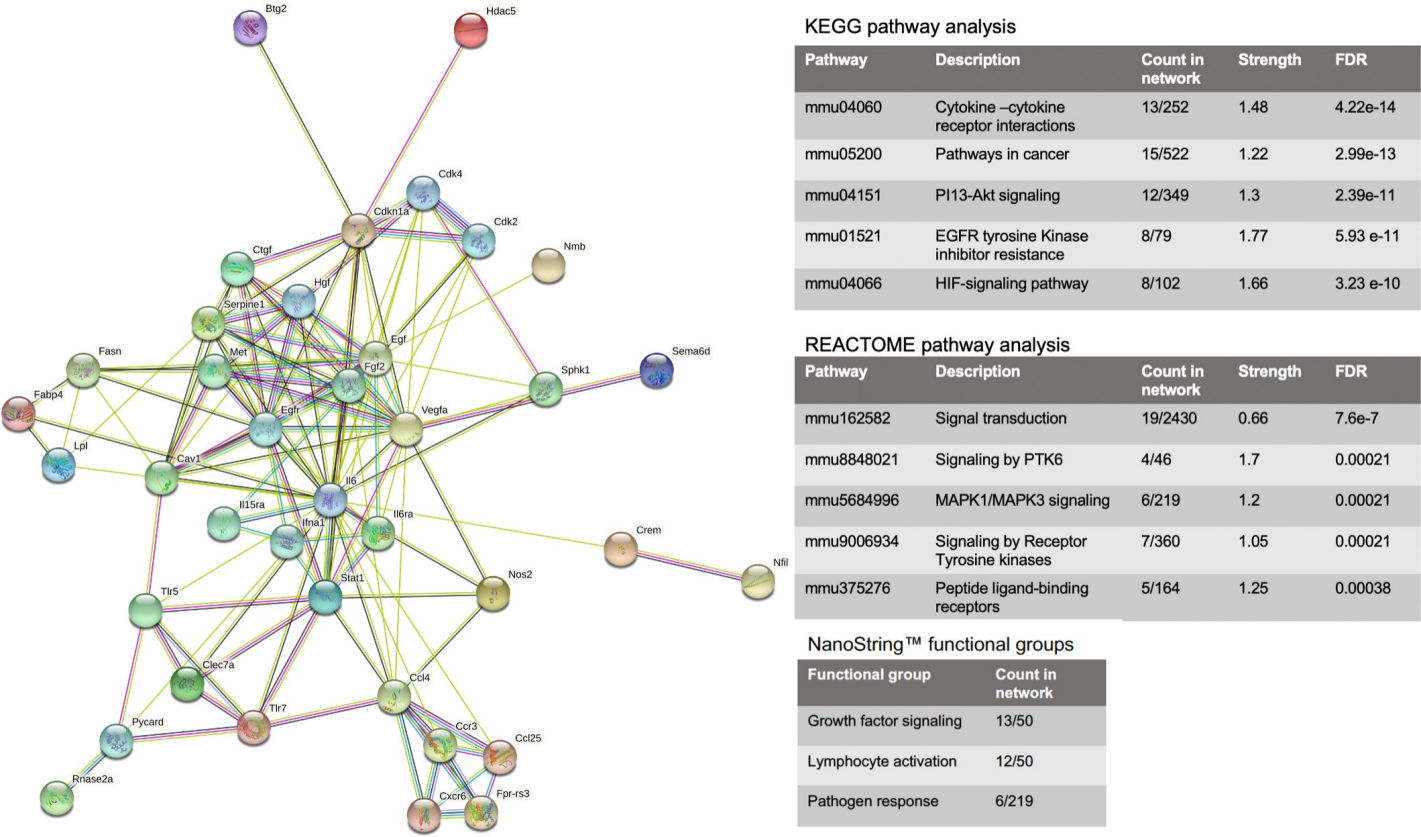
Protein-protein interaction network. PPI network was generated using the top hits of both joint and muscle tissues combined. The cut off criteria included at least a 1.3 FC and a *P value < 0.02. Circles represent genes and lines represent the interaction of proteins between genes. Line colour represents evidence of interaction between the proteins. Top hits were selected based on lowest FDR. Results for both KEGG and REACTOME pathways are shown. Top three functional groups as identified with NanoString™ annotations are also shown.

To analyse the relationship between the DEGs modulated by PPS treatment, a protein-protein interaction (PPI) network was created using the STRING database. To understand the overall mechanisms of PPS, the PPI network was generated using modulated genes from both joint and muscle, two clinically relevant tissues for the pathobiology of CHIKV infection. A total of 50 DEGs consisting of 24 upregulated genes and 26 downregulated genes from joint and muscle tissues were inputted. The cut off criteria for the top DEGs were as follows: at least a 1.3 FC and a *P value < 0.02. After removing the isolated and partially connected nodes, a final, complex network of DEGs was constructed (Fig 7). Within the final PPI network, a total of 38 nodes remained with an average node degree of 7.05. The PPI enrichment ****P value was <1.0e-16, indicating the identified proteins are at least partially biologically connected, as a group.

Factors including fibroblast growth factor (FGF) and IL-6 serve as central nodes with high levels of interaction, indicating critical roles in the effects seen in the PPS-treatment of CHIKV-infected untreated mice. Nodes including STAT1, IFNα1, epidermal growth factor (EGF) and vascular endothelial growth factor A (VEGFA) demonstrated a high degree of interaction, which again suggest key roles in the effects seen with PPS treatment during CHIKV infection. Overall, the PPI network provides fundamental insights into the key targets of PPS treatment of CHIKV-induced inflammatory disease.

Results of key KEGG and REACTOME pathways were also generated using STRING analysis. The top five results, based on FDR and identified via the KEGG pathway were cytokine-cytokine receptor interactions, pathways in cancer, phosphatidylinositol 3-kinase PI3-AKT signalling, epidermal growth factor receptor (EGFR), tyrosine kinase inhibitor resistance and hypoxia inducible factor (HIF) signalling pathway. For REACTOME, the top 5 pathways included signal transduction pathways, signalling by tyrosine-protein kinase 6 (PTK6), mitogen-activated protein kinase 1 and 3 (MAPK1/MAPK3) signalling, signalling by receptor tyrosine kinases and peptide-ligand-binding receptors (FDR < 0.0004; Fig 7).

Top DEGs were also sorted using functional group analysis. NanoString™ provides functional annotations to classify DEGs of a specific panel into biological process categories. The top three functional groups included: growth factor signalling (*Il-15ra*, *Il-1β*, *Cdkn1a*, *Il-6ra*, *Egf*, tyrosine-protein kinase Met (*Met)*, nuclear factor of activated T cell 2 (*Nfatc2*), tyrosine-protein phosphatase non-receptor type 14 (*Ptpn14*), *Stat1*, *Vegfa*, histone deacetylase 5 (*Hdac5*), Ras-specific guanine nucleotide-releasing factor 2 (*Rasgrf2*) and Fgf2), lymphocyte activation (*Clec7a*), adenosine A_3_ receptor (*Adora3*), *Cdkn1a*, *Fcer1a*, formyl peptide receptor-related sequence 3 (*Fpr-rs3*), apoptosis-associated speck-like protein containing a CARD (*Pycard*; ASC), *Egf*, *Fgf2*, *Nfatc2*, Semaphorins 6D (*Semad6*), *Il-1β* and *Rasgrf2*) and pathogen response (*Crem*, KRAB-A domain containing 1 (*Krba1*), ribonuclease 2 (*Rnase2*), nitric oxide synthase 2 (*Nos2*), *Ccl4*, *Il-1β*, *Pycard*, proteoglycan 2 (*Prg2*) and *Trex1*).

## Discussion

This study provides novel information into the effects of PPS treatment in mice infected with CHIKV. PPS is a repurposed semisynthetic glycosaminoglycan that has been shown to be well-tolerated in both oral and injectable forms. Recently, injectable PPS (iPPS) was administered to a cohort of RRV infected patients with no serious adverse events reported [15]. We have demonstrated that CHIKV-infected untreated mice develop the classical rheumatic features of local inflammation as observed by foot and joint swelling and histological evidence of arthritis as seen by cellular infiltrates, tenosynovitis and myositis. Furthermore, we have shown for the first time, that CHIKV-infected untreated mice experience a prolonged and significant decrease in grip strength, an important rheumatological function test [28].

Low grip strength is a predictor and a criterion for frailty, functional decline and physical malfunction. Decreases in grip and pinch strength are a strong indicator of functional disability in patients with rheumatic conditions such as RA [29]. Importantly, it has been shown that patients with post-CHIKV rheumatism have impaired balance, gait and reduced grip strength when compared to non-CHIKV patients of similar age [30]. Our findings demonstrate that this adult mouse model is not only useful to examine the pathogenesis of CHIKV but can also be an important tool to study functional assays related to CHIKV disease which may have a translational benefit to clinic. Furthermore, when mice were treated with PPS, not only was local inflammation reduced, but grip strength was also protected. At peak disease, only CHIKV-infected untreated animals presented loss in grip strength when compared to their baseline values. PPS treatment not only attenuated local inflammation in this animal model but was shown to prevent limb dysfunction as evidenced by reduced foot swelling and grip strength respectively.

Histological analysis indicated that PPS as a treatment for CHIKV arthritis resulted in a decrease in infiltrating cells in the calcaneal joints and muscles at peak disease. It has been shown that *in vitro* incubation of PPS on STRO-1^+^ immuno-selected mesenchymal stem cells (MSCs) lead to increased viability and chondrogenic differentiation whilst suppressing osteogenesis [31]. In accordance with the literature, our study showed that mice treated with PPS appeared to have better myocyte regeneration and less cartilage damage when compared to CHIKV-infected untreated animals (S3 Fig). More recently, the same group demonstrated that PPS enhances chondrogenesis by altering genetic and proteomic signatures leading to increased proliferation of MSCs [32]. It is possible that similar mechanisms are responsible for the protection against muscle and cartilage damage seen in CHIKV-infected PPS-treated mice.

Circulating biomarkers were examined at peak disease, and significant decreases in key chemokines were noted. Reductions in CCL2, CCL7 and CCL12 could further explain the reduced number of cellular infiltrates seen in the CHIKV-infected PPS-treated mice. CCL2 has been well characterised in atherosclerosis [33] and arthritis [34] development. In a recent meta-analysis study, CCL2 was identified as an important marker for the progression of osteoarthritis (OA) with the authors concluding that it may serve as a potential biomarker for the diagnosis of OA [35]. CCL2 recruits mostly monocytes and to a lesser extent, memory T cells and dendritic cells to sites of inflammation. Furthermore, a recent study showed that CCL2 and its receptor CCR2 also contribute to the regulation of pain-related behaviour [36]. The contribution of CCL2 to the debilitating pain in alphaviral arthritis has yet to be examined. However, it is of interest to note that the use of an CCL2 inhibitor, Bindarit, or a CCL2 antibody were shown to alleviate alphaviral induced arthropathies [37, 38].

CCL7 and CCL12 have been shown to have strong chemotaxis functions thereby contributing to the influx of immune cells to the site of inflammation. CCL7 has been shown to increase the synovial fluid of patients with OA [39] whereas CCL12 has known functions in regulating joint formation and limb ossification during development [40]. In a mouse model of OA, it was shown that CCL12 levels increase in both bone and cartilage during early phases of development [41] making it an interesting therapeutic target towards the prevention of arthritis. Furthermore, our data also showed a significant decrease in the chemokine CXCL1 (KC). CXCL1 is responsible for the recruitment of neutrophils to the site of infection [42]. Neutrophils have been shown to be involved in the development of arthritis in most experimental animal models [43]. It was shown that a reduction in neutrophils can attenuate disease in several models of arthritis including adjuvant [44], collagen [45] and collagen antibody-induced arthritis [46]. Taken together, the reduction seen in circulating serum biomarkers may reflect the attenuated disease state seen in CHIKV-infected PPS-treated mice. CXCL13 (BCA-1) was also shown to be increased with PPS-treatment in CHIKV-infected PPS-treated mice. It is well recognised that CXCL13 is involved in the recruitment of B cells to the synovial tissue in RA, where they exert pathogenic functions [47]. Interestingly, it has been recently described that CXCL13 can also attenuate inflammation [48]. Although its exact role has not been elucidated in the context of PPS treatment in CHIKV-infected mice, it is plausible that its overexpression could also contribute to the amelioration of clinical disease.

It has previously been shown that PPS causes a reduction in inflammatory markers such as IL-1β, TNF-α and IL-6 as well as inhibition of the complement system [49, 50]. Studies on canine chondrocytes *in vitro* have shown that PPS can affect a number of signalling pathways including the P38, extracellular-signal-regulated kinase (ERK) [51], inducible nitric oxide synthase (iNOS), c-Jun and HIF-1α [52]. Furthermore, in primary human osteocytes, mRNA and protein levels of the pain mediator, nerve growth factor (NGF) was also shown to be reduced in the presence of PPS [53]. For Ross River virus (RRV) induced arthritis, it was speculated that inhibition of rheumatic disease with PPS treatment was due to a reduction in IL-6 and CCL2 [14]. To better understand how PPS is reducing clinical signs of CHIKV disease in mice, we used the NanoString™ technology to profile the expression of 754 targeted genes in both joint and muscle tissues. Importantly, the nCounter® myeloid innate immune panel used in this study identified some of the same RNA targets as previously described by using RNA-Seq analysis [27] thereby validating this novel approach towards profiling CHIKV disease. When comparing mock with CHIKV-infected untreated joint tissues (7 d.p.i.) the key DEGs included *Cxcl10* (IP-10), *Cxcl9*, *Fcgr4*, *Ccl7*, *Ccl2*, *Ccr5*, *Ccl8*, *Fcgr1* and *Gzma* (S6A Fig). As for muscle, our top DEGs were *Cxcl9*, *Fcgr4*, *Gzma*, *Cxcl10*, *Stat1*, *Ccl5*, natural killer cell granule protein 7 (*Nkg7*), and *Cd274* (S6B Fig).

Our data shows that *in vivo*, PPS activates pathways specific to each tissue (Figs 5 and 6; S3 and S4 Tables). This is not uncommon as pharmacodynamics of drugs varies within the body and therefore can trigger different responses. Among the most significantly modulated genes, some were of particular interest due to their clinical relevance for arthritis. These include *Ctgf*, *Cdkn1a*, *Nfil3*, *Stat1*, *Fabp4*, *Crem*, *Clec7a*, *Il-1β*, *Mertk*, *Trex1*, *Btg2*, *Serpine1*, *Ccl25* and *Olr1*. *Nfil3* was seen to be upregulated in both tissues. NFIL3 binds to activating transcription factor (ATF) sites in many cellular and viral promoters. It plays a role in insulin-like growth factor 1 (IGF1) receptor signalling and nuclear factor kappa-light-chain-enhancer of activated B cells (NF-κβ) signalling. Interestingly, ATF-6 is part of the unfolded protein response (UPR) response and it is well documented that CHIKV can activate the ‘activating transcription factor 6’ (ATF-6) and IRE-1 branches of the UPR response [54]. NFIL3 also impairs Treg cell function through the downregulation of forkhead box P3 (Foxp3) expression [55]. Studies in knockout mice show that in the absence of *Nfil3*, susceptibility to arthritis is enhanced [56]. *Nfil3* upregulation observed in our study may suggest a possible role for PPS in these pathways.

It has been shown that mice deficient in *Mertk* demonstrated more cartilage proteoglycan depletion, and cartilage and bone erosion than wild type mice in a RA model of infection using CIA. These mice also had increased amounts of infiltrating cells into the synovium [57]. This is particularly interesting as PPS-treated mice also saw decreased cartilage damage and a reduction in the number of infiltrating cells suggesting that the upregulation of MERTK seen in the joints may help protect against CHIKV pathogenesis.

*Ccl25* was upregulated in PPS-treated CHIKV joints and is known to be involved in mobilisation of MSCs. As mentioned above, it has already been shown that PPS can prime and help MSC proliferation. Furthermore, CCL25 was shown, *in vivo*, to contribute to the attenuation of cartilage degeneration during OA [58].

*Fgf2* was found to be downregulated in muscle tissues of mice treated with PPS. FGF2 is increased in RA patients and was shown to be correlated with grade of bone erosion [59, 60]. Furthermore, FGF2 causes proteoglycan depletion in cartilage as seen in our CHIKV infections. Other reports suggest that damaged caused by FGF2 during RA can also be attributed to its ability to induce osteoclastogenesis [60].

The other clinically relevant genes, collectively, display a number of functions in arthritis. The regulation of these genes may prevent disease by acting on cell proliferation and invasion (*Cdkn1A* [61] and *Btg2* [62]), suppressing inflammation (*Fgf2* [63]), osteoclastogenesis (*Vegfa* [64]), angiogenesis (*VegfA*, *Fgf2*) and cytokine and chemokine signalling (*Stat1* [65], *Il-1β* [66, 67] and *Cxcr6* [68, 69]).

Many of these identified genes (*Cxcr6*, *Crem*, *Clec7A*, *Fpr-rs3* and *Nfil3*) have known involvement with T cells leading to the hypothesis that T cell regulation may be an important mechanism of action of PPS. This is interesting as T cell-mediated immunity is known to contribute to the immunopathogenicity of CHIKV [70, 71]. Furthermore, some of these molecules like IL-1β, HDAC5 and OLR1 (LOX-1) have already been flagged as potential therapeutic targets for RA [72–74] strengthening their importance in arthropathies.

To explore how PPS might be reducing the inflammation and CHIKV-induced functional decline, both KEGG and REACTOME pathway analysis was performed. Identified pathways included those known to be involved in various types of arthritis. For example, one study examined the biological pathways involved in RA and OA by KEGG analysis and found that cytokine-cytokine receptor interactions, PI13-AKT signalling and pathways in cancer were all important when comparing to normal controls [75]. Another study identifying pathways and genes associated with synovitis in OA also noted the importance of pathways in cancer and cytokine-cytokine receptor interaction [76]. The PI13-AKT and the MAPK1/MAPK3 signalling pathways identified by KEGG and REACTOME analyses are noteworthy as activated FGF signalling plays a pivotal role in sustaining stem cells capabilities through the activation of RAS-MAPK, PI3K/AKT, phospholipase C gamma (PLCγ) and STAT [77]. Previous studies have already established that PPS plays an important role inhibiting MAPK (via ERK) pathways [51]. Furthermore, the Ras-ERK and PI3K-mTOR pathways interact to regulate each other and co-regulate downstream functions by cross-inhibition or cross-activation [78]. One reason for this is that ERK can phosphorylate several members of the core signalling pathways as well as many other effector proteins.

We further classified the key target genes into functional groups using annotations provided by NanoString™. The top 3 functional groups identified for our target genes were growth factor signalling, lymphocyte activation and pathogen response. Growth factors are essential regulators in the development, homeostasis and pathogenesis of the joint making them interesting therapeutic candidates for the treatment of RA and OA. One method to repair damaged articular cartilage, consists of stimulating MSCs with growth factors [79]. Many including TGF-β, BMP-2, BMP-7, IGF-1 and FGF-18 are current therapeutic targets being investigated for potential clinical use [80]. However, other members of these growth factor families like those belonging to the transforming growth factor-β superfamily (TGF-β), fibroblast growth factor family (FGF), insulin-like growth factor-I (IGF-1), and platelet-derived growth factor (PDGF) may also be of interest for clinical applications. Interestingly, the growth factor functional group had the greatest number of our top DEGs (13/50) meaning it is the group which saw the most modulated genes from PPS treatment. Moreover, it is known that PPS can stimulate MSCs *in vitro* [31, 32]. Perhaps this mechanism occurs via one of the newly identified growth factors and leads to decreased cartilage damage during CHIKV infection.

The next group in importance that encompasses our top DEGs was lymphocyte activation. This group contained 12/50 DEGs from this study. As previously mentioned, T cells play an important role in CHIKV pathogenesis therefore finding a therapeutic drug that targets lymphocytes could alleviate alphaviral disease. Joint inflammation and synovitis are severe in mice infected with CHIKV. Treatment of PPS reduced inflammation and protected cartilage from damage. Furthermore, T cell infiltrates are also important mediators of inflammation in RA and to a lesser extent OA [81]. PPS treatment in RA and OA patients may prove beneficial as well as for diseases with strong lymphocyte inflammation like lymphocytic colitis.

Out of 50 PPS modulated DEGs, 9 belonged to the pathogen response group. It is interesting to note that a pathogen response signature has been identified in peripheral blood of RA patients [82]. The signatures observed included increased expression of type 1 IFNs, decreased gamma-delta (γδ) gene expression, reduced transcript levels of HLA class II molecules and reduced transcriptional activity. Taken together, these signalling pathways and functional groups may play the most important roles in the mechanism of action of PPS during CHIKV infection. These findings also reinforce the notion that PPS is an effective treatment for a variety of arthropathies including RA, OA and alphaviral induced arthritis.

Overall, our study has demonstrated for the first time that PPS is an effective treatment against CHIKV-induced arthritis through investigation of a number of parameters. Mice treated with PPS did not display muscle weakness as measured by grip strength and had significantly reduced foot swelling. Furthermore, a decreased number of infiltrates, significantly less cartilage damage and increase muscle repair were some positive outcomes of PPS treatment. Through the use of bioinformatic analysis, we gained insight into potential mechanisms of action of PPS during viral arthritis. Key genes that could explain reduction in the severity of disease signs include *Nfil3*, *Mertk*, *Ccl25* and *Fgf2*. Furthermore, PPS mechanism of action appears to be linked to three KEGG pathways also seen to be important in arthropathies. These include pathways in cytokine-cytokine receptor interactions, pathways in cancer and PI13-AKT signalling. When examining the critical functional groups, PPS was shown to exert effect on growth factor signalling, lymphocyte activation and pathogen response, all which likely contribute to the amelioration of CHIKV-induced arthritic disease.

## Supporting information

S1 Fig Weights did not vary significantly during PPS treatment. C57BL/6 mice were infected s.c. with 10^4^ PFU CHIKV or PBS alone and received daily injections of PPS-treatment or mock-treatment with PBS. Weight change was assessed daily during the course of the study. No significant differences were observed between any of the groups (n = 15 mice/group from 0–7 d.p.i. and n = 5 animals/group from 8–21 d.p.i.). *Two-Way ANOVA with a Tukey’s post-test*.
(TIF)

S2 Fig CHIKV disease was resolved by day 21 post infection. C57BL/6 mice were infected s.c. with 10^4^ PFU CHIKV or PBS alone and received daily injections of PPS-treatment or mock-treatment with PBS. Mice were sacrificed at 21 d.p.i. and tissues collected, fixed and stained with H&E for histological analysis. The number of cellular infiltrates seen in the muscles of each group was not significantly different confirming disease resolution. However, mice that were treated with PPS displayed less muscle fibre damage when compared to CHIKV-infected mock-treated animals. Slides were scanned with the Aperio Scan Scope XT digital slide scanner. A representative image from each group of mice is shown. Images are representatives of 5 mice per group. Scale bar represents 100 μm.
(TIF)

S3 Fig PPS treatment of CHIKV-infected mice aids in myocyte regeneration. C57BL/6 mice were infected s.c. with 10^4^ PFU CHIKV or PBS alone and received daily injections of PPS-treatment or mock-treatment with PBS. Mice were sacrificed at 7 d.p.i. and tissues collected, fixed and stained with H&E for histological analysis. Mice that were treated with PPS displayed improved myocyte regeneration as seen by infiltrating repair monocytes. Regenerating myocytes are characterized by centrally aligned nuclei and dark-stained cytoplasm (indicated by arrows). Slides were scanned with the Aperio Scan Scope XT digital slide scanner. A representative image from each group of mice is shown. Images are representatives of 5 mice per group. Scale bar represents 60 μm.
(TIF)

S4 Fig PPS is not antiviral. To confirm that the method of action of PPS at acute infection (7 d.p.i.) is not due to an antiviral effect, C57BL/6 mice were infected s.c. with 10^4^ PFU CHIKV and received daily injections of PPS-treatment or mock-treatment with PBS. Mice were sacrificed at 7 d.p.i., and tissues were collected, and RNA extracted. 1 ug of RNA was reversed transcribed to cDNA using Tetro™ cDNA Synthesis Kit (Meridian Bioscience). CHIKV genome copy numbers (GCN) quantification was done using the following primers for nsP2 F: 5’—CCGAAAGGAAACTTCAAAGCAACT- 3’ and R: 5’ -CAGATGCCCGCCATTATTGATG—3’. The SensiFAST™ SYBR® No-ROX kit (Meridian Bioscience) was used according to the manufacturer’s instructions. Cycling conditions were: 3 min at 95°C, followed by 40 cycles of 5 s at 95°C, 10 s at 58°C and 20 s at 72°C. Purified plasmid DNA containing full-length Réunion Island CHIKV isolate LR2006-OPY1 genome was serially diluted and used as standards. Viral genome copy numbers were calculated based on the amount of DNA in the standards (g) and the size of the plasmid. Cq values were plotted using Graphpad Prism and the corresponding GCN values for each sample were extrapolated from the standard curve. RNA analysed was from 5 animals/group. Statistical analysis to compare the CHIKV-infected untreated group to the CHIKV-infected PPS-treated group was performed using a *One-Way ANOVA with a Tukey’s post-test*. No statistical significance was found.
(TIF)

S5 Fig Serum chemokine and cytokine levels that were not altered. As part of the Bio-Plex Pro Mouse Chemokine Panel 33-Plex, chemokine and cytokine levels of mock, PPS alone (PPS), CHIKV-infected untreated (CHIKV) and CHIKV-infected PPS-treated (CHIKV/PPS) mice were assessed at 7 d.p.i. (peak disease). All values are presented as mean pg/mL ± SEM of 5 mice per group. *One-Way ANOVA with a Tukey’s post-test* was used but showed no statistical significance between groups.
(TIF)

S6 Fig DEGs regulated in joint (A) and muscle tissues (B) at peak disease during CHIKV infection. Gene expression analysis of RNA was performed using the commercially available NanoString™ nCounter® mouse Myeloid Innate Immunity gene expression panel. Differentially expressed genes in CHIKV-infected untreated (CHIKV) mice compared to mock animals (n = 3 mice/group) were identified at 7 d.p.i. (peak disease). Data were graphed as volcano plots and heat maps for key DEGs of (A) joint or (B) muscle tissues. Genes had at least a 3-fold change and a **P value ≤ 0.01.
(TIF)

S1 Table Table of DEGs up (A) and down (B) regulated in joint tissue at peak disease. Gene expression analysis of RNA was performed using the commercially available NanoString™ nCounter® mouse Myeloid Innate Immunity gene expression panel. Differentially expressed genes found in the joints of CHIKV-infected untreated mice were compared to those of mock animals on 7 d.p.i. (peak disease) and listed in a table (n = 3 mice/group). Genes had at least a 3-fold change and a **P value ≤ 0.01. Asterisks depicts genes common to both joint and muscle tissues.
(TIF)

S2 Table Table of DEGs up (A) and down (B) regulated in muscle tissue at peak disease. Gene expression analysis of RNA was performed using the commercially available NanoString™ nCounter® mouse Myeloid Innate Immunity gene expression panel. Differentially expressed genes found in the quadriceps of CHIKV-infected untreated mice were compared to those of mock animals on 7 d.p.i. (peak disease) and listed in a table (n = 3 mice/group). These genes had at least a 3-fold change and a **P value ≤ 0.01. Asterisks depicts genes common to both joint and muscle tissues.
(TIF)

S3 Table Table of up-regulated (A) and down-regulated DEGs (B) in joints at peak disease during PPS treatment. Gene expression analysis of RNA was performed using the commercially available NanoString™ nCounter® mouse Myeloid Innate Immunity gene expression panel. Differentially expressed genes found in the joints of CHIKV-infected PPS-treated mice were compared to those of CHIKV-infected untreated animals on 7 d.p.i. (peak disease) and listed in a table (n = 3 mice/group). Top genes chosen had a FC >1.3 or FC < -1.3 and a *P value < 0.02. Asterisks depicts genes common to both joint and muscle tissues.
(TIF)

S4 Table Table of DEGs up (A) and down (B) regulated in muscle tissue at peak disease during PPS treatment. Gene expression analysis of RNA was performed using the commercially available NanoString™ nCounter® mouse Myeloid Innate Immunity gene expression panel. Differentially expressed genes found in quadriceps of CHIKV-infected PPS-treated mice compared to those of CHIKV-infected untreated animals were identified on 7 d.p.i. (peak disease) and listed in a table (n = 3 mice/group). Top genes chosen had a FC >1.3 or FC < -1.3 and a *P value < 0.02. Asterisks depicts genes common to both joint and muscle tissues.
(TIF)
