# Supplementary figures and images for: Pentosan polysulfate sodium prevents functional decline in chikungunya infected mice by modulating growth factor signalling and lymphocyte activation
Source: PLoS One. 2021 Sep 7;16(9):e0255125. doi: 10.1371/journal.pone.0255125 (PMC8423248; doi:10.1371/journal.pone.0255125)

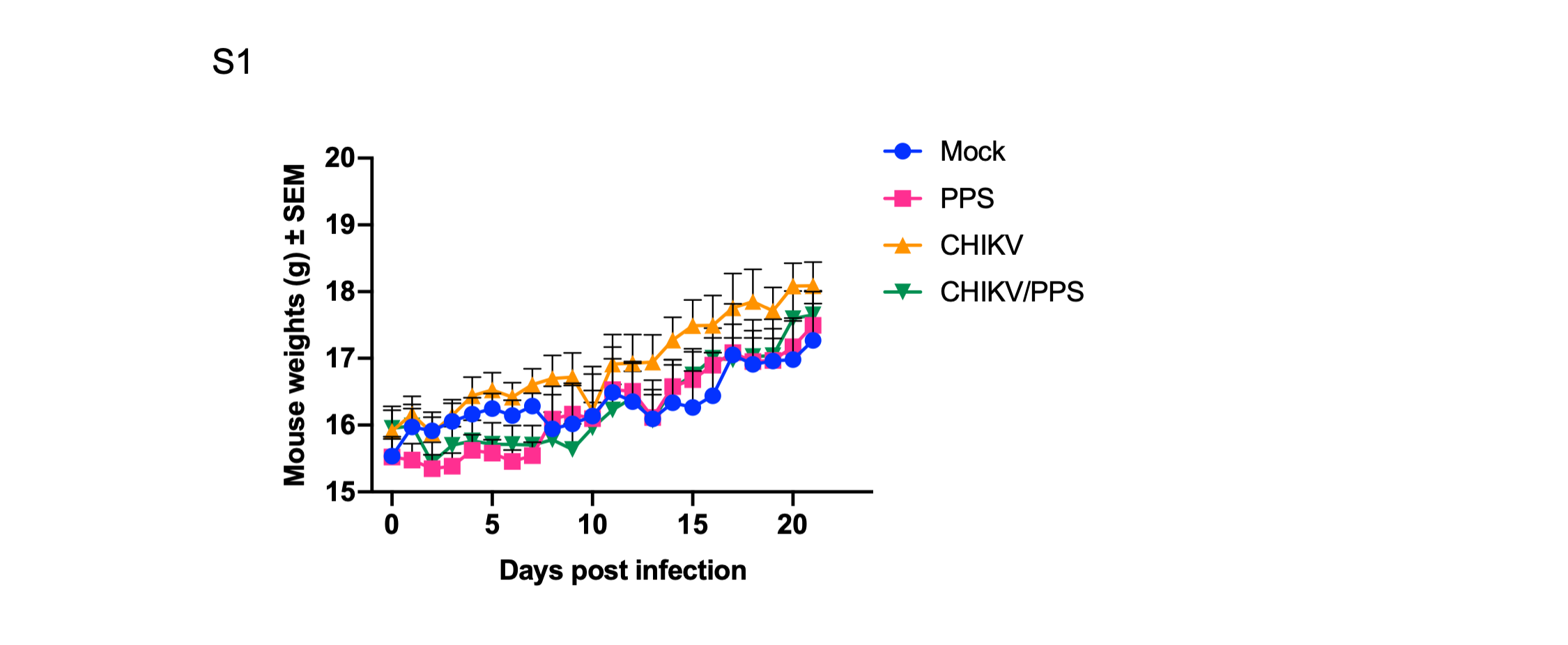

Supplement: S1 Fig — C57BL/6 mice were infected s.c. with 104 PFU CHIKV or PBS alone and received daily injections of PPS-treatment or mock-treatment with PBS. Weight change was assessed daily during the course of the study. No significant differences were observed between any of the groups (n = 15 mice/group from 0–7 d.p.i. and n = 5 animals/group from 8–21 d.p.i.). Two-Way ANOVA with a Tukey’s post-test. (TIF) [file pone.0255125.s001.tif]

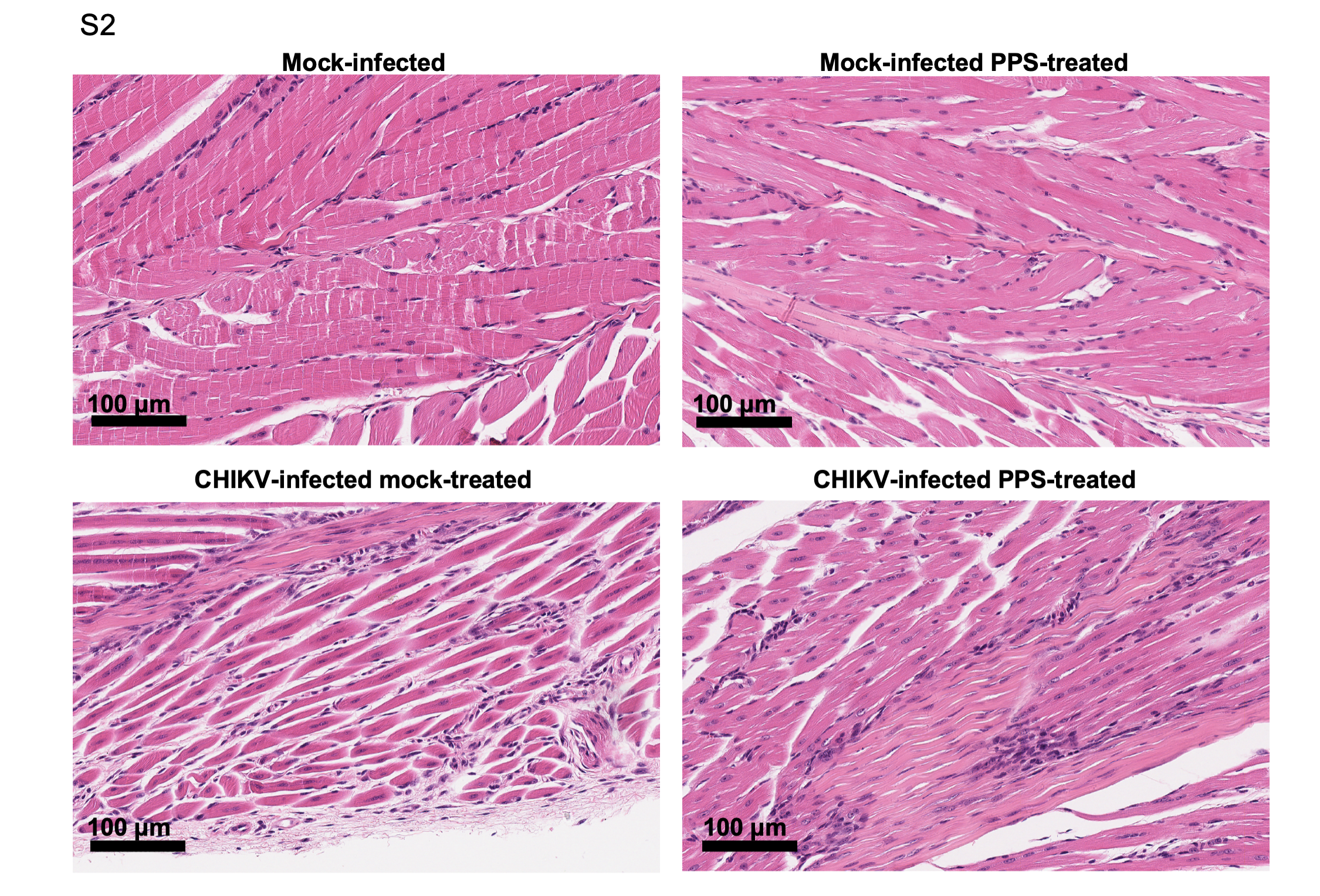

Supplement: S2 Fig — C57BL/6 mice were infected s.c. with 104 PFU CHIKV or PBS alone and received daily injections of PPS-treatment or mock-treatment with PBS. Mice were sacrificed at 21 d.p.i. and tissues collected, fixed and stained with H&E for histological analysis. The number of cellular infiltrates seen in the muscles of each group was not significantly different confirming disease resolution. However, mice that were treated with PPS displayed less muscle fibre damage when compared to CHIKV-infected mock-treated animals. Slides were scanned with the Aperio Scan Scope XT digital slide scanner. A representative image from each group of mice is shown. Images are representatives of 5 mice per group. Scale bar represents 100 μm. (TIF) [file pone.0255125.s002.tif]

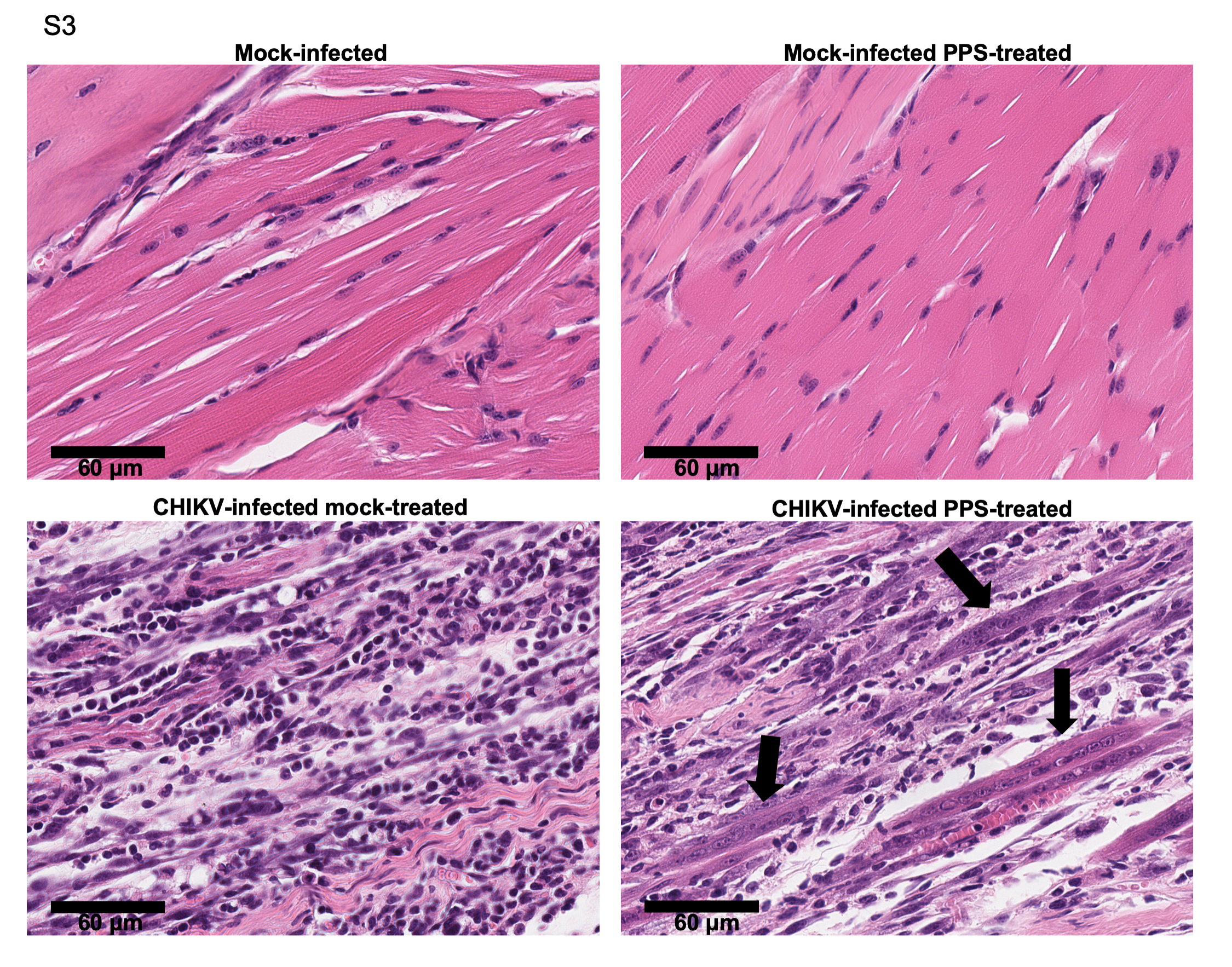

Supplement: S3 Fig — C57BL/6 mice were infected s.c. with 104 PFU CHIKV or PBS alone and received daily injections of PPS-treatment or mock-treatment with PBS. Mice were sacrificed at 7 d.p.i. and tissues collected, fixed and stained with H&E for histological analysis. Mice that were treated with PPS displayed improved myocyte regeneration as seen by infiltrating repair monocytes. Regenerating myocytes are characterized by centrally aligned nuclei and dark-stained cytoplasm (indicated by arrows). Slides were scanned with the Aperio Scan Scope XT digital slide scanner. A representative image from each group of mice is shown. Images are representatives of 5 mice per group. Scale bar represents 60 μm. (TIF) [file pone.0255125.s003.tif]

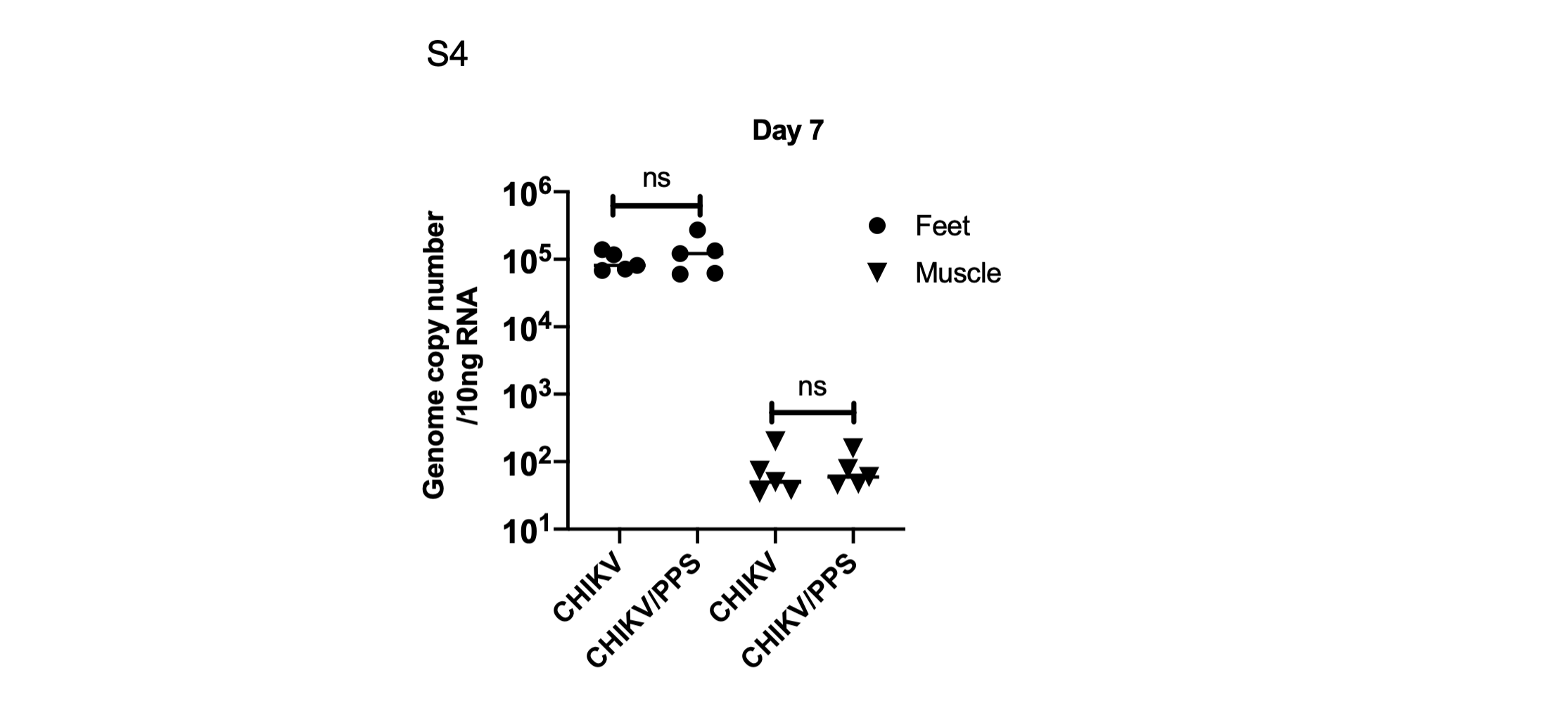

Supplement: S4 Fig — To confirm that the method of action of PPS at acute infection (7 d.p.i.) is not due to an antiviral effect, C57BL/6 mice were infected s.c. with 104 PFU CHIKV and received daily injections of PPS-treatment or mock-treatment with PBS. Mice were sacrificed at 7 d.p.i., and tissues were collected, and RNA extracted. 1 ug of RNA was reversed transcribed to cDNA using Tetro™ cDNA Synthesis Kit (Meridian Bioscience). CHIKV genome copy numbers (GCN) quantification was done using the following primers for nsP2 F: 5’—CCGAAAGGAAACTTCAAAGCAACT- 3’ and R: 5’ -CAGATGCCCGCCATTATTGATG—3’. The SensiFAST™ SYBR® No-ROX kit (Meridian Bioscience) was used according to the manufacturer’s instructions. Cycling conditions were: 3 min at 95°C, followed by 40 cycles of 5 s at 95°C, 10 s at 58°C and 20 s at 72°C. Purified plasmid DNA containing full-length Réunion Island CHIKV isolate LR2006-OPY1 genome was serially diluted and used as standards. Viral genome copy numbers were calculated based on the amount of DNA in the standards (g) and the size of the plasmid. Cq values were plotted using Graphpad Prism and the corresponding GCN values for each sample were extrapolated from the standard curve. RNA analysed was from 5 animals/group. Statistical analysis to compare the CHIKV-infected untreated group to the CHIKV-infected PPS-treated group was performed using a One-Way ANOVA with a Tukey’s post-test. No statistical significance was found. (TIF) [file pone.0255125.s004.tif]

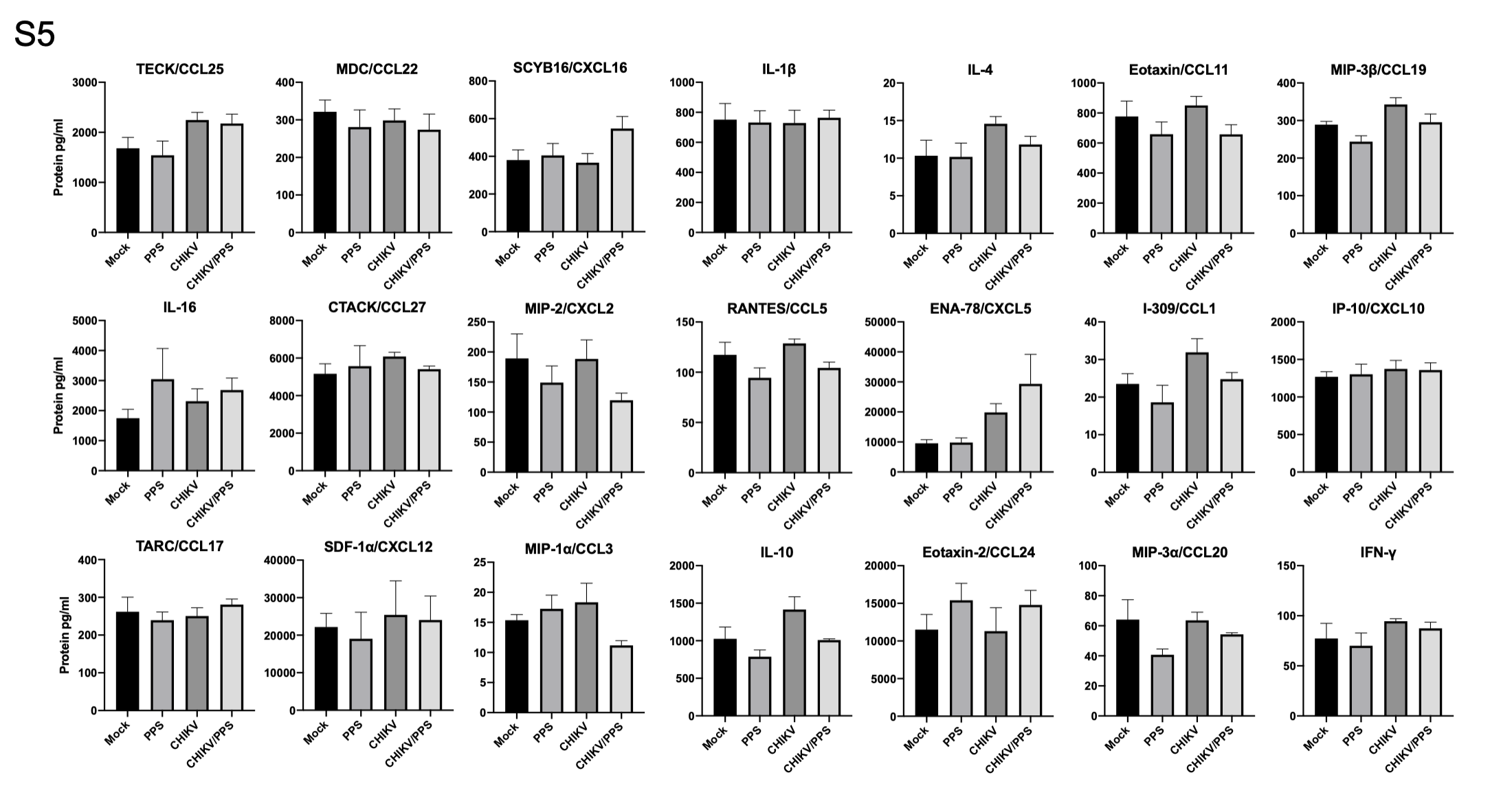

Supplement: S5 Fig — As part of the Bio-Plex Pro Mouse Chemokine Panel 33-Plex, chemokine and cytokine levels of mock, PPS alone (PPS), CHIKV-infected untreated (CHIKV) and CHIKV-infected PPS-treated (CHIKV/PPS) mice were assessed at 7 d.p.i. (peak disease). All values are presented as mean pg/mL ± SEM of 5 mice per group. One-Way ANOVA with a Tukey’s post-test was used but showed no statistical significance between groups. (TIF) [file pone.0255125.s005.tif]

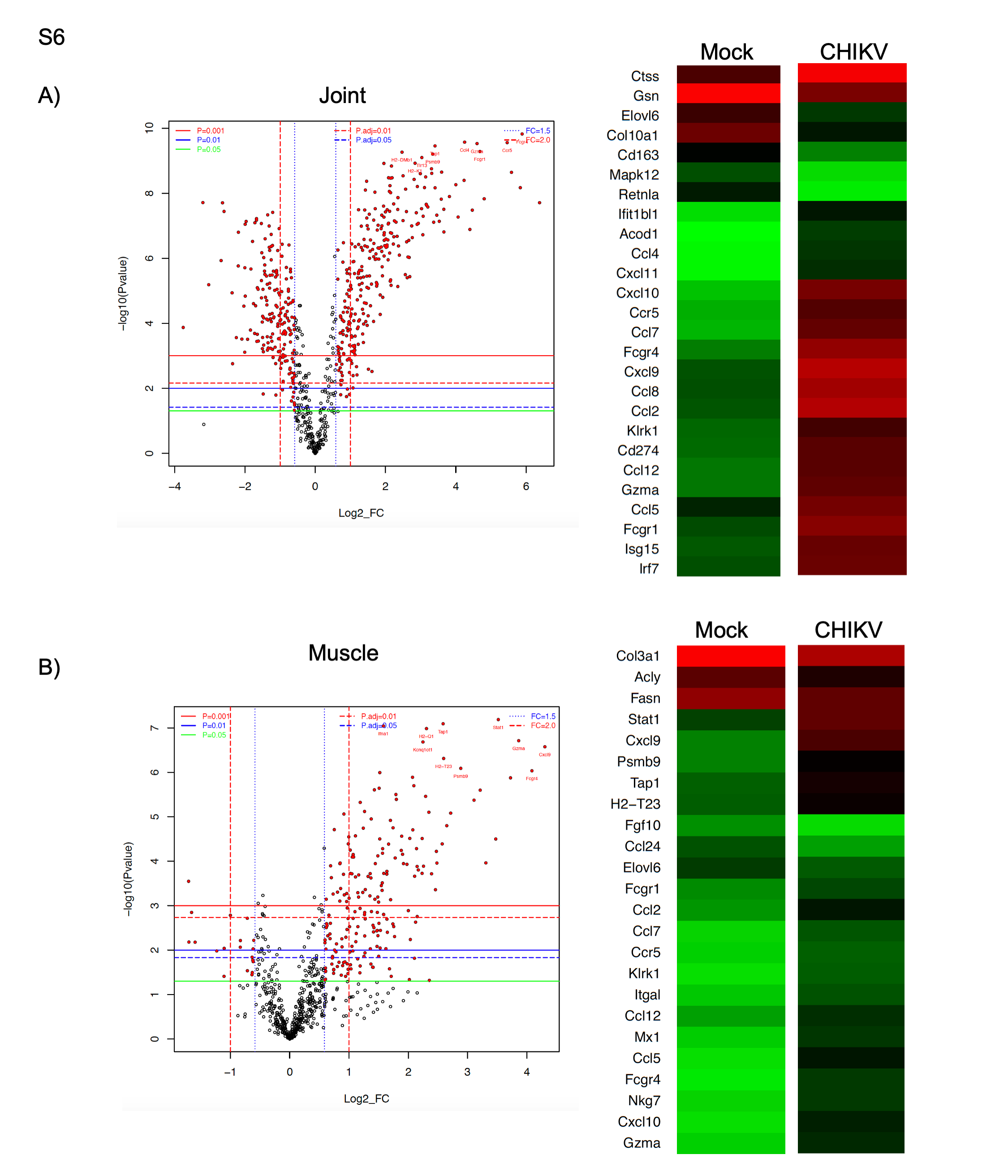

Supplement: S6 Fig — DEGs regulated in joint (A) and muscle tissues (B) at peak disease during CHIKV infection. Gene expression analysis of RNA was performed using the commercially available NanoString™ nCounter® mouse Myeloid Innate Immunity gene expression panel. Differentially expressed genes in CHIKV-infected untreated (CHIKV) mice compared to mock animals (n = 3 mice/group) were identified at 7 d.p.i. (peak disease). Data were graphed as volcano plots and heat maps for key DEGs of (A) joint or (B) muscle tissues. Genes had at least a 3-fold change and a **P value ≤ 0.01. (TIF) [file pone.0255125.s006.tif]

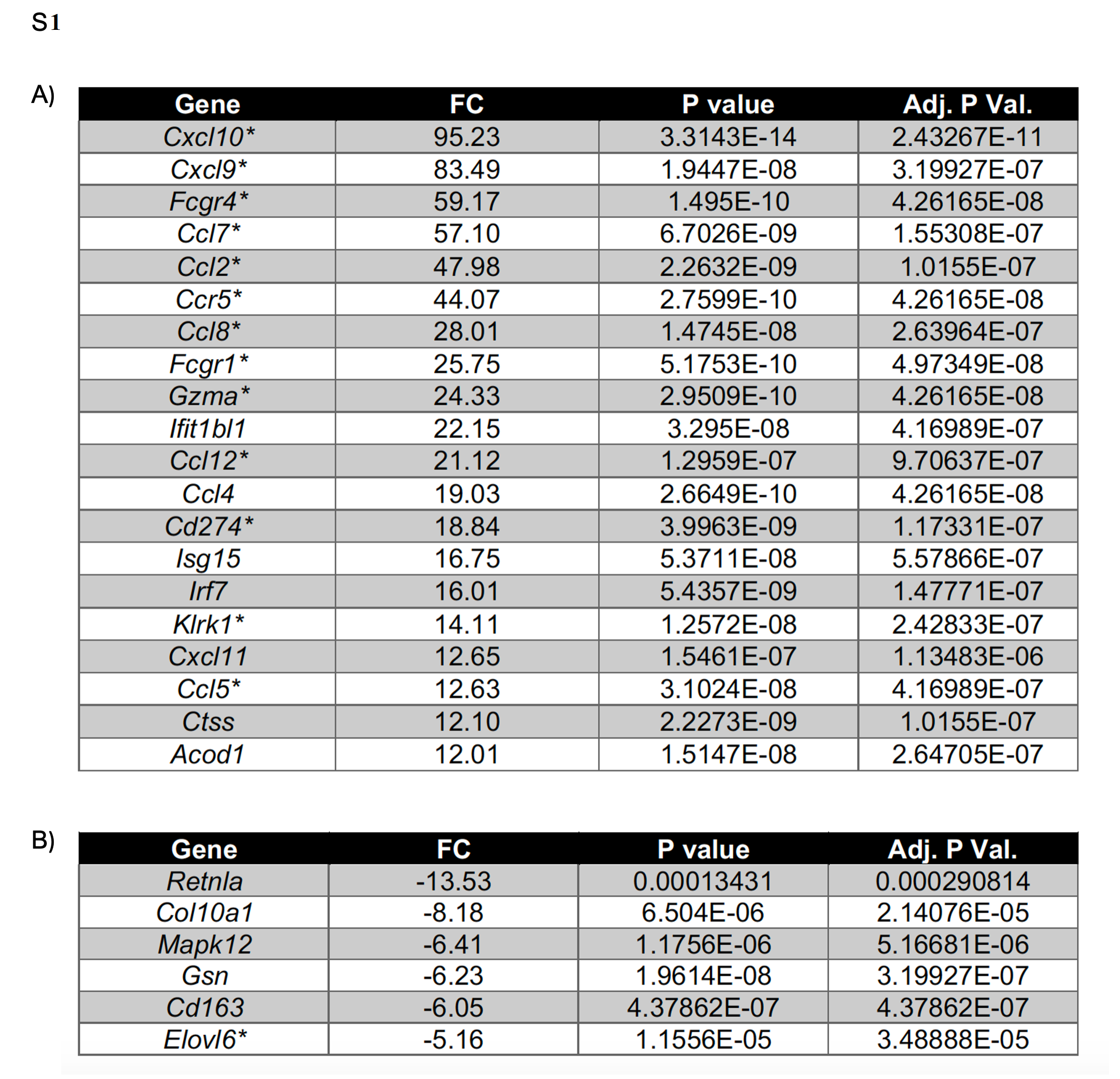

Supplement: S1 Table — Table of DEGs up (A) and down (B) regulated in joint tissue at peak disease. Gene expression analysis of RNA was performed using the commercially available NanoString™ nCounter® mouse Myeloid Innate Immunity gene expression panel. Differentially expressed genes found in the joints of CHIKV-infected untreated mice were compared to those of mock animals on 7 d.p.i. (peak disease) and listed in a table (n = 3 mice/group). Genes had at least a 3-fold change and a **P value ≤ 0.01. Asterisks depicts genes common to both joint and muscle tissues. (TIF) [file pone.0255125.s007.tif]

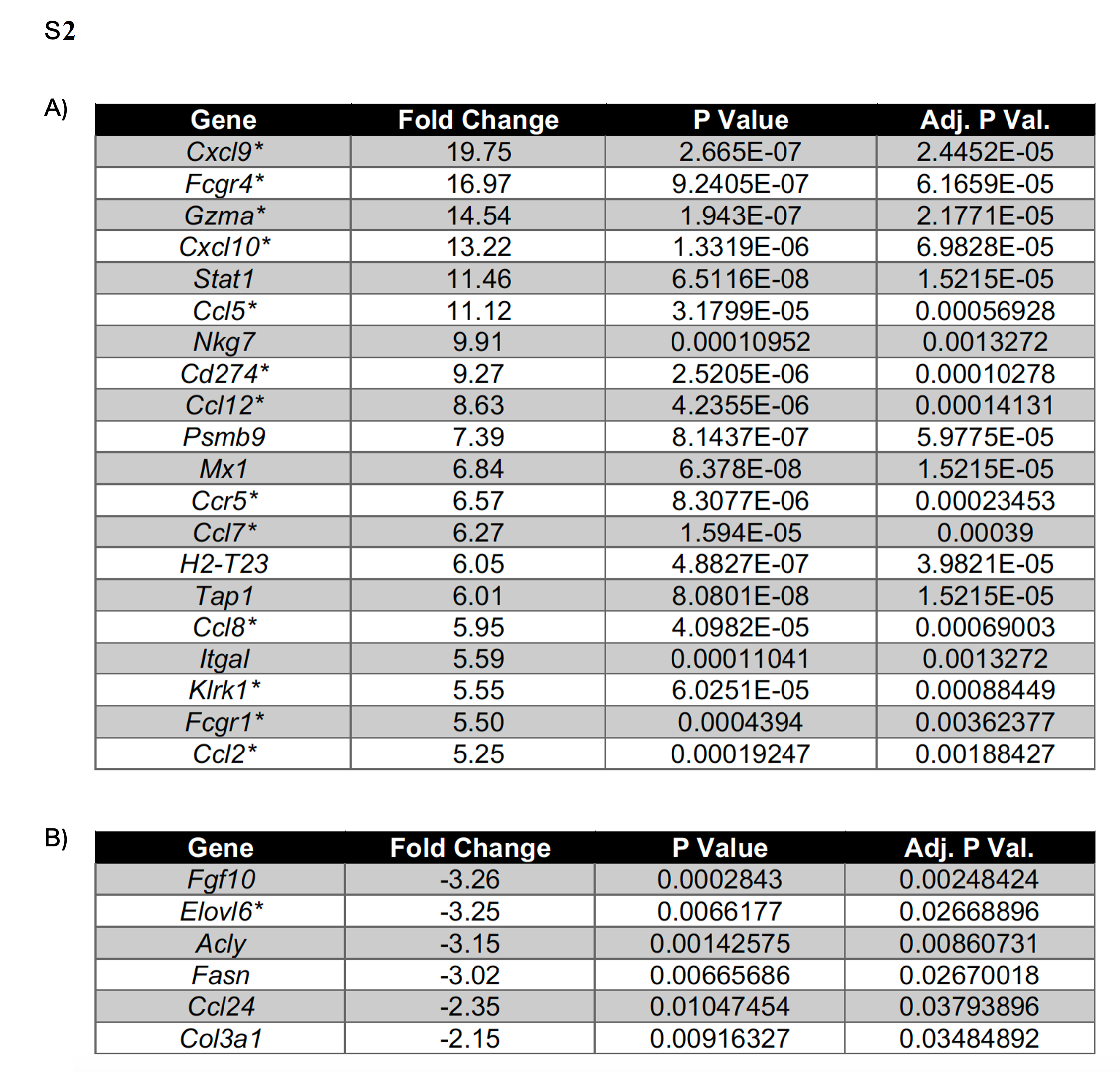

Supplement: S2 Table — Table of DEGs up (A) and down (B) regulated in muscle tissue at peak disease. Gene expression analysis of RNA was performed using the commercially available NanoString™ nCounter® mouse Myeloid Innate Immunity gene expression panel. Differentially expressed genes found in the quadriceps of CHIKV-infected untreated mice were compared to those of mock animals on 7 d.p.i. (peak disease) and listed in a table (n = 3 mice/group). These genes had at least a 3-fold change and a **P value ≤ 0.01. Asterisks depicts genes common to both joint and muscle tissues. (TIF) [file pone.0255125.s008.tif]

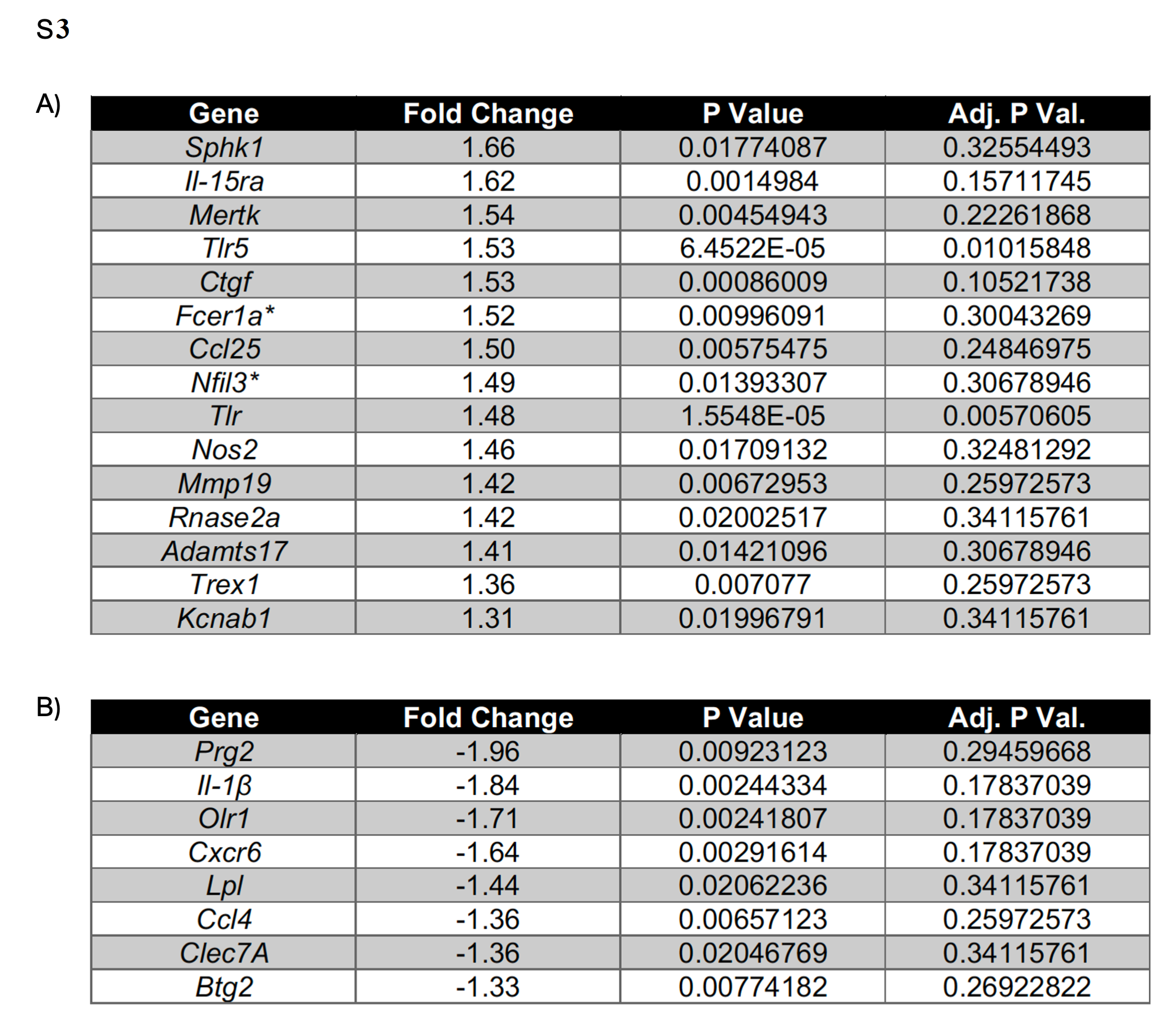

Supplement: S3 Table — Table of up-regulated (A) and down-regulated DEGs (B) in joints at peak disease during PPS treatment. Gene expression analysis of RNA was performed using the commercially available NanoString™ nCounter® mouse Myeloid Innate Immunity gene expression panel. Differentially expressed genes found in the joints of CHIKV-infected PPS-treated mice were compared to those of CHIKV-infected untreated animals on 7 d.p.i. (peak disease) and listed in a table (n = 3 mice/group). Top genes chosen had a FC >1.3 or FC < -1.3 and a *P value < 0.02. Asterisks depicts genes common to both joint and muscle tissues. (TIF) [file pone.0255125.s009.tif]

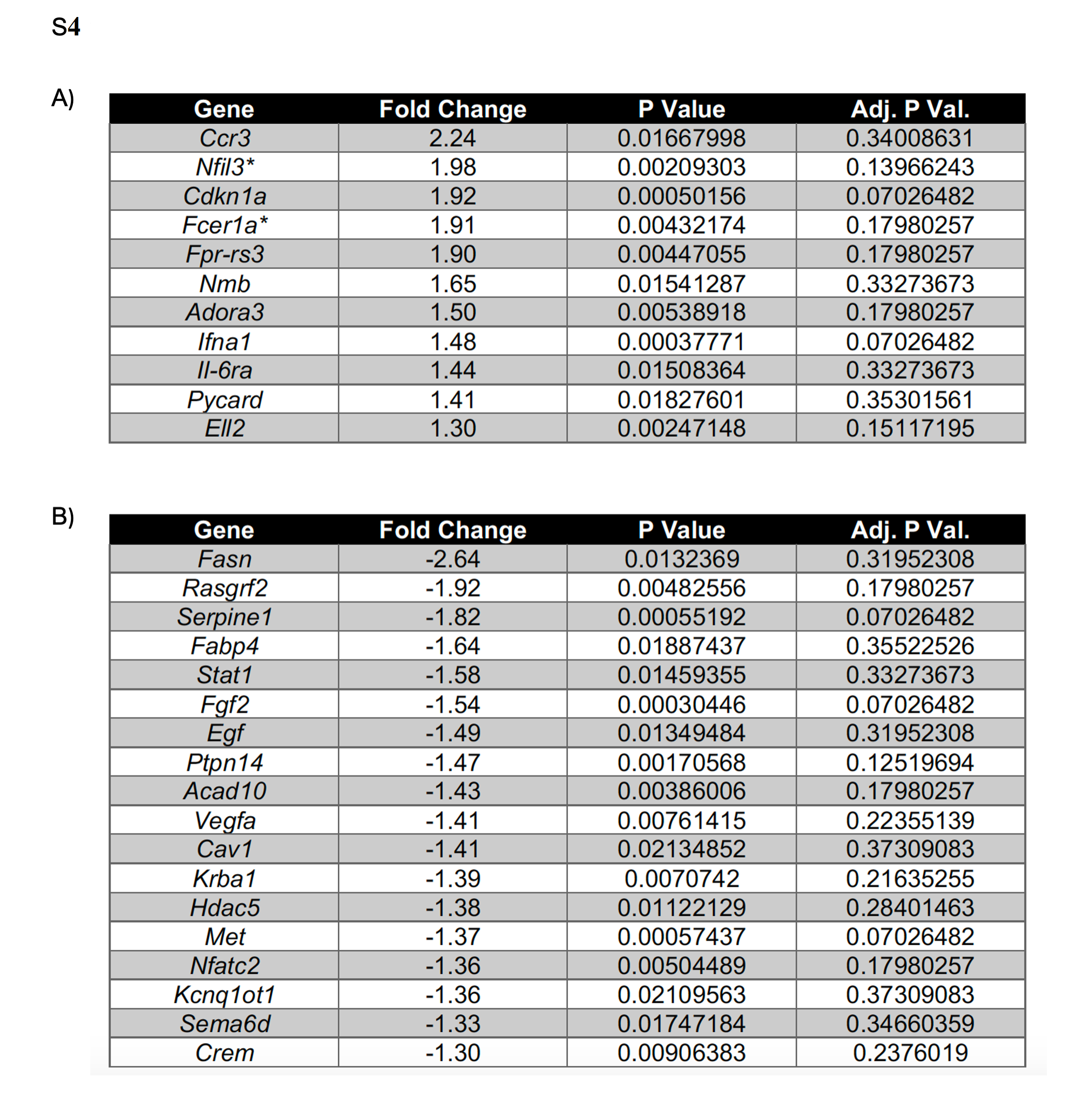

Supplement: S4 Table — Table of DEGs up (A) and down (B) regulated in muscle tissue at peak disease during PPS treatment. Gene expression analysis of RNA was performed using the commercially available NanoString™ nCounter® mouse Myeloid Innate Immunity gene expression panel. Differentially expressed genes found in quadriceps of CHIKV-infected PPS-treated mice compared to those of CHIKV-infected untreated animals were identified on 7 d.p.i. (peak disease) and listed in a table (n = 3 mice/group). Top genes chosen had a FC >1.3 or FC < -1.3 and a *P value < 0.02. Asterisks depicts genes common to both joint and muscle tissues. (TIF) [file pone.0255125.s010.tif]
